# The enzyme mechanism of patchoulol synthase

**DOI:** 10.3762/bjoc.18.2

**Published:** 2022-01-03

**Authors:** Houchao Xu, Bernd Goldfuss, Gregor Schnakenburg, Jeroen S Dickschat

**Affiliations:** 1 Kekulé-Institute of Organic Chemistry and Biochemistry, University of Bonn, Gerhard-Domagk-Straße 1, 53121 Bonn, Germany; 2 Department of Chemistry, University of Cologne, Greinstraße 4, 50939 Cologne, Germany; 3 Institute of Inorganic Chemistry, University of Bonn, Gerhard-Domagk-Straße 1, 53121 Bonn, Germany

**Keywords:** biosynthesis, DFT calculations, enzyme mechanisms, isotopes, terpenes

## Abstract

Different mechanisms for the cyclisation of farnesyl pyrophosphate to patchoulol by the patchoulol synthase are discussed in the literature. They are based on isotopic labelling experiments, but the results from these experiments are contradictory. The present work reports on a reinvestigation of patchoulol biosynthesis by isotopic labelling experiments and computational chemistry. The results are in favour of a pathway through the neutral intermediates germacrene A and α-bulnesene that are both reactivated by protonation for further cyclisation steps, while previously discussed intra- and intermolecular hydrogen transfers are not supported. Furthermore, the isolation of the new natural product (2*S*,3*S*,7*S*,10*R*)-guaia-1,11-dien-10-ol from patchouli oil is reported.

## Introduction

**Figure 1:**
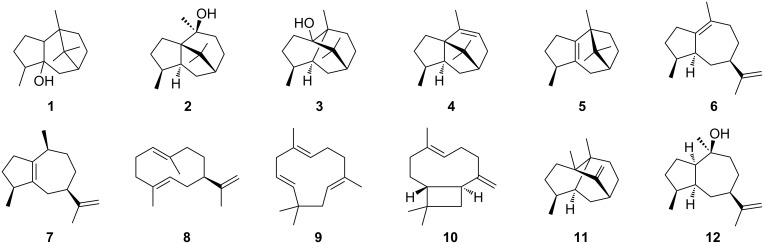
Initially assigned structures for patchoulol by Treibs (**1**) and by Büchi (**2**). Structures of patchoulol (**3**) and side products of patchoulol synthase (**4**–**12**).

Patchouli oil, the essential oil of the shrub *Pogostemon cablin*, has a pleasant woody odour and is of high economic value for the perfumery and cosmetics industries. It is mainly composed of sesquiterpenes with patchoulol as the main compound (ca. 40%) [1–2]. Pure patchoulol is a crystalline material that has first been described by Gal in 1869 [3]. Its planar structure was initially described as that of compound **1** (Figure 1) by Treibs [4], and later reassigned to structure **2** based on a total synthesis from camphor by Büchi [5]. Because of an unexpected rearrangement this structural assignment was still erroneous, and the correct structure **3** was finally established by X-ray analysis of its chromic acid diester [6]. The patchoulol synthase (PTS) has been purified from plant leaves and shown to convert farnesyl diphosphate (FPP) into compound **3** and several biogenetically related terpene hydrocarbons including α-patchoulene (**4**), β-patchoulene (**5**), α-bulnesene (**6**) and α-guaiene (**7**) (Figure 1) [7]. The enzyme was subsequently made available by cDNA gene cloning, revealing germacrene A (**8**), α-humulene (**9**), (*E*)-β-caryophyllene (**10**), seychellene (**11**) and pogostol (**12**) as further side products [8].

**Scheme 1:**
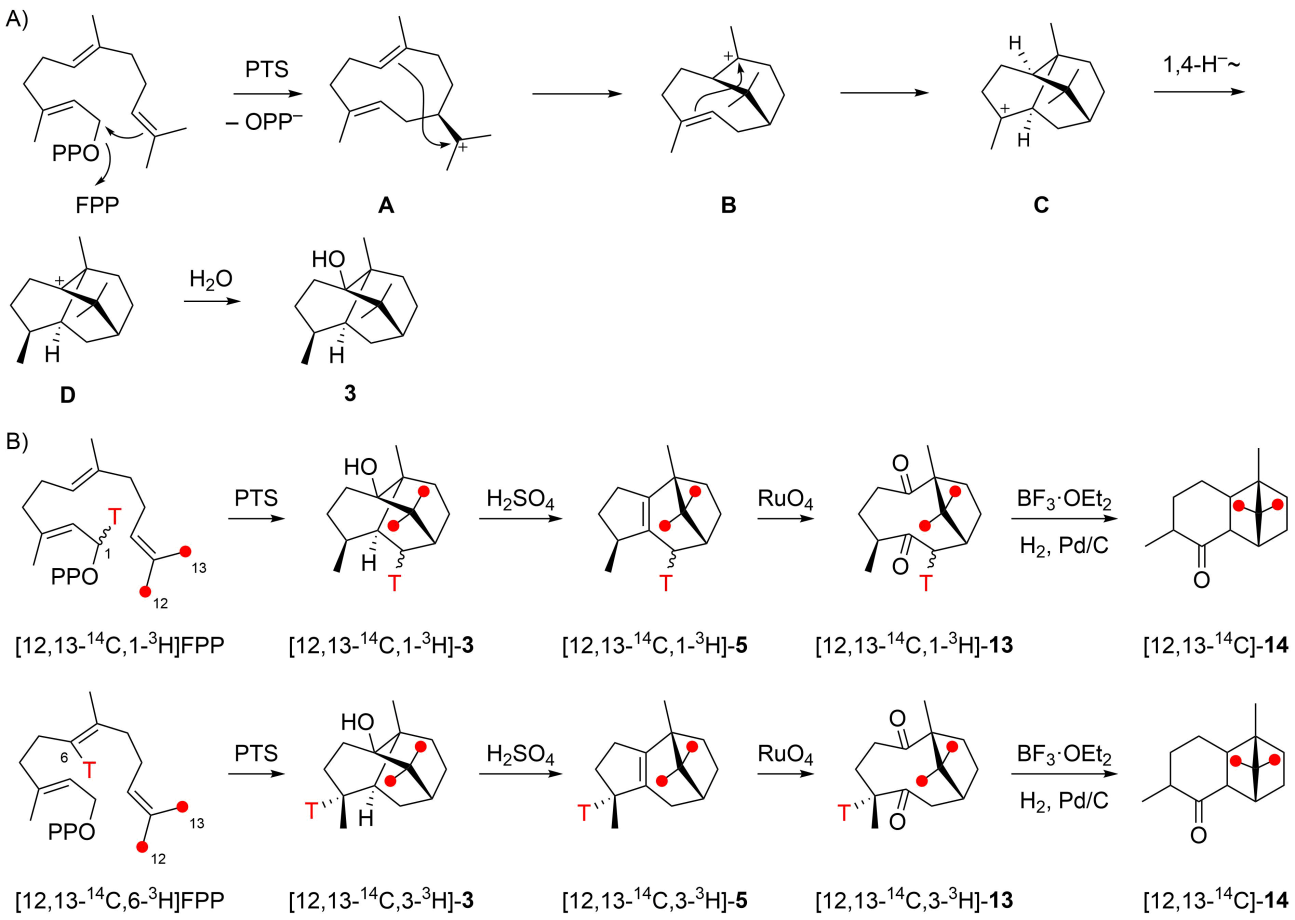
Biosynthesis of patchoulol (part I). A) Cyclisation mechanism from FPP to **3** as suggested by Croteau et al., and B) labelling experiments reported in the same study [9]. For all sesquiterpenes in this study the carbon numbering follows that of FPP to indicate the (proposed) biosynthetic origin of each carbon. Red dots indicate ^14^C-labelled carbons.

The biosynthetic mechanism of the formation of compound **3** was investigated by several groups through isotopic labelling experiments. In 1987, Croteau et al. have suggested a pathway through 1,10-cyclisation of FPP to the (*E*,*E*)-germacradienyl cation (**A**), followed by direct cyclisation reactions to **B** and **C**, a 1,4-hydride shift to **D** and capture with water to yield **3** (Scheme 1A) [9]. This mechanism was supported by radioactive labelling experiments with [12,13-^14^C,1-^3^H]FPP and [12,13-^14^C,6-^3^H]FPP, whose enzymatic conversion with PTS into **3** proceeded with full retainment of the labelling in both cases (Scheme 1B). Subsequent chemical degradation through acid catalysed conversion into **5**, oxidative cleavage to the diketone **13**, BF_3_∙OEt_2_ mediated ring closure by aldol reaction and catalytic hydrogenation gave **14**. For both experiments a full retainment of labelling was reported for all intermediates until **13**, while a loss of tritium was observed for **14** with both substrates. From these experiments it was concluded that the hydrogen H6 must migrate into another position, as realised by the 1,4-hydride shift from **C** to **D**. The loss of ^3^H in the experiment with [12,13-^14^C,1-^3^H]FPP was expected for the aldol reaction of **13**, but is more difficult to understand in the experiment with [12,13-^14^C,6-^3^H]FPP. In this case the loss of ^3^H was explained by an exchange against ^1^H during catalytic hydrogenation [9].

**Scheme 2:**
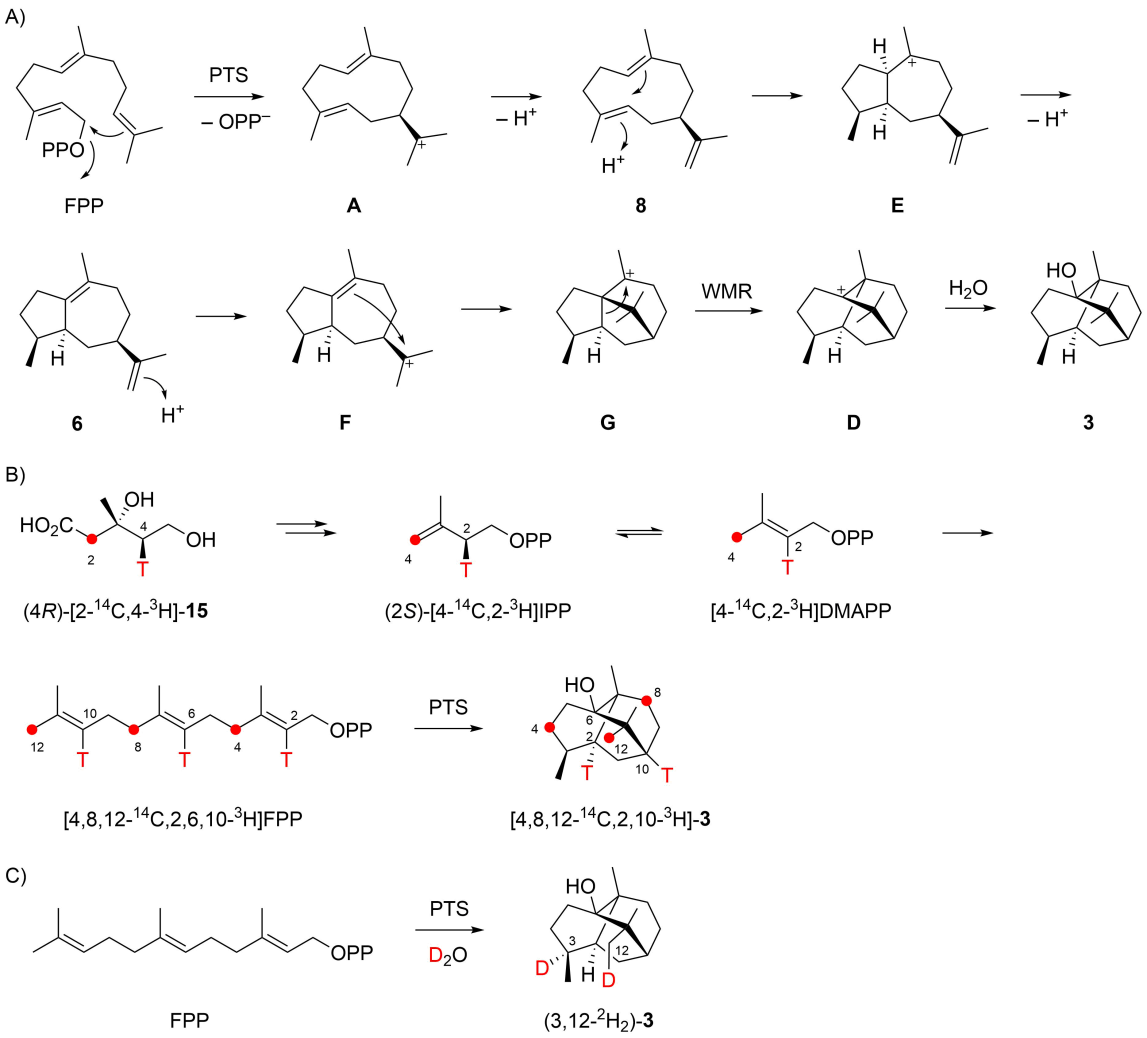
Biosynthesis of patchoulol (part II). A) Cyclisation mechanism from FPP to **3** as suggested by Akhila et al., and B) labelling experiments reported in the same study [10]. C) Labelling experiment by Ekramzadeh et al. [13]. Red dots indicate ^14^C-labelled carbons. WMR = Wagner–Meerwein rearrangement.

One year later, Akhila et al. proposed an alternative biosynthetic mechanism that also starts with a cyclisation of FPP to **A** (Scheme 2A) [10], but then a subsequent deprotonation to **8**, an important neutral intermediate in the biosynthesis of many sesquiterpenes [11], is assumed. A reprotonation-induced cyclisation leads to **E** that is again deprotonated to **6**, followed by another reprotonation to **F**, cyclisation to **G** and Wagner–Meerwein rearrangement to **D**, the same final intermediate as suggested by Croteau. This mechanism was supported by feeding experiments with (4*R*)-[2-^14^C,4-^3^H]mevalonic acid (**15**) that is converted through IPP and DMAPP into FPP (Scheme 2B). According to the FPP biosynthesis as established by Cornforth and co-workers, these reactions should proceed with full retainment of all labellings [12]. For isolated **3** a loss of one of the three ^3^H atoms was reported that is explainable by the deprotonation step from **E** to **6** [10], but contradicts the retainment of this hydrogen as reported by Croteau [9]. Further support for Akhila’s mechanism was provided by Ekramzadeh et al., who observed the uptake of two deuterium atoms at C3 and C12 in an incubation of FPP with PTS in deuterium oxide buffer that explain the reprotonations of the neutral intermediates **8** and **6** (Scheme 2C) [13].

**Scheme 3:**
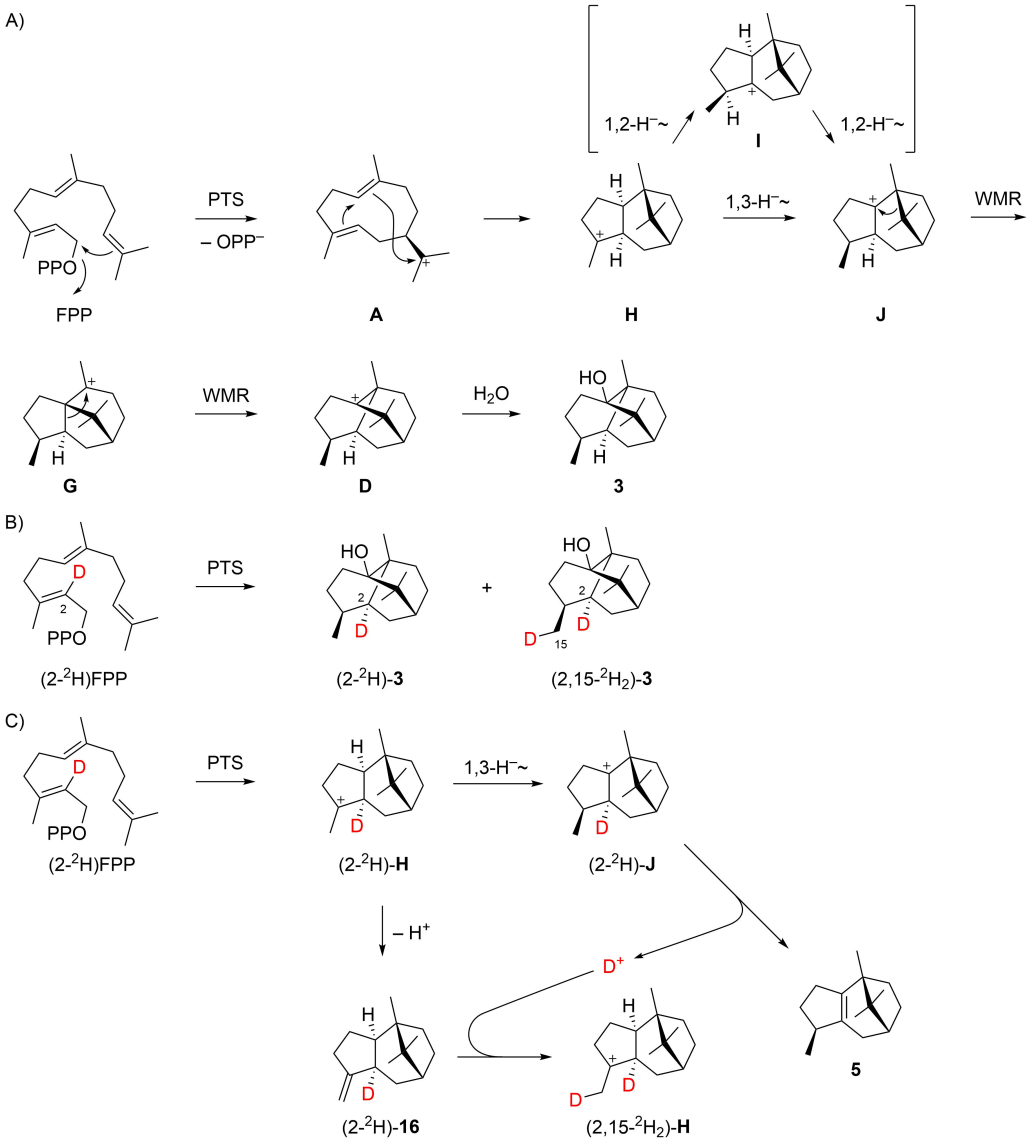
Biosynthesis of patchoulol (part III). A) Cyclisation mechanism from FPP to **3** as suggested by Faraldos et al., B) labelling experiments reported in the same study, and C) proposed intermolecular proton transfers to explain the formation of doubly deuterated products from a singly deuterated substrate [14]. WMR = Wagner–Meerwein rearrangement.

In 2010, Faraldos et al. published a third mechanism that also starts with a cyclisation of FPP to **A** (Scheme 3A) [14]. Similar to Croteau’s mechanism, **A** is directly further cyclised to **H**, followed by a 1,3-hydride shift to **J** (equivalent to the 1,4-hydride migration from **C** to **D** in Scheme 1A), and a Wagner–Meerwein rearrangement to **G**. The final steps are identical to those in Akhila’s mechanism (Scheme 2A). This work also reported on a labelling experiment with (2-^2^H)FPP that was enzymatically converted with PTS with incorporation of deuterium at C2 of **3** (Scheme 3B). This result ruled out that the 1,3-hydride shift from **H** to **J** must be replaced by two sequential 1,2-hydride transfers via **I**, but cannot discriminate between the Croteau’s and Akhila’s mechanistic alternatives. In addition, the formation of doubly labelled (2,15-^2^H_2_)-**3** from (2-^2^H)FPP was reported, which was explained by an unusual intramolecular deuterium transfer. Herein, the deuteron is released from (2-^2^H)-**J** in the deprotonation step to **5** (or other enzyme products losing the same hydrogen in the terminal deprotonation). Deprotonation of (2-^2^H)-**H** was suggested to produce the unknown sesquiterpene (2-^2^H)-**16** that may take up the deuteron released in the formation of **5** (and similar compounds) to give (2,15-^2^H_2_)-**H** (Scheme 3C).

Notably, none of the proposed mechanisms in Schemes 1–3 can explain the reported results from all labelling experiments and some of the reported findings are even contradictory. For this reason, we have reinvestigated the enzyme mechanism of PTS in isotopic labelling experiments through methods recently developed in our laboratory that make use of ^13^C and ^2^H-substituted terpene precursors, and by DFT calculations. The general strategy in these experiments is to use substrates or substrate combinations so that deuterium migrations end at ^13^C-labelled carbons, resulting in triplet signals in the ^13^C NMR spectra [15–16]. Moreover, deuterium atoms ending in neighbouring positions of ^13^C-labelled carbons become evident from slight upfield shifted ^13^C NMR signals. These experiments and the DFT calculations were not only carried out in a way to gain support for one mechanism, but also to disprove some of the earlier reports in order to resolve the contradictions in the literature.

## Results and Discussion

### Absolute configurations of patchoulol and pogostol

**Figure 2:**
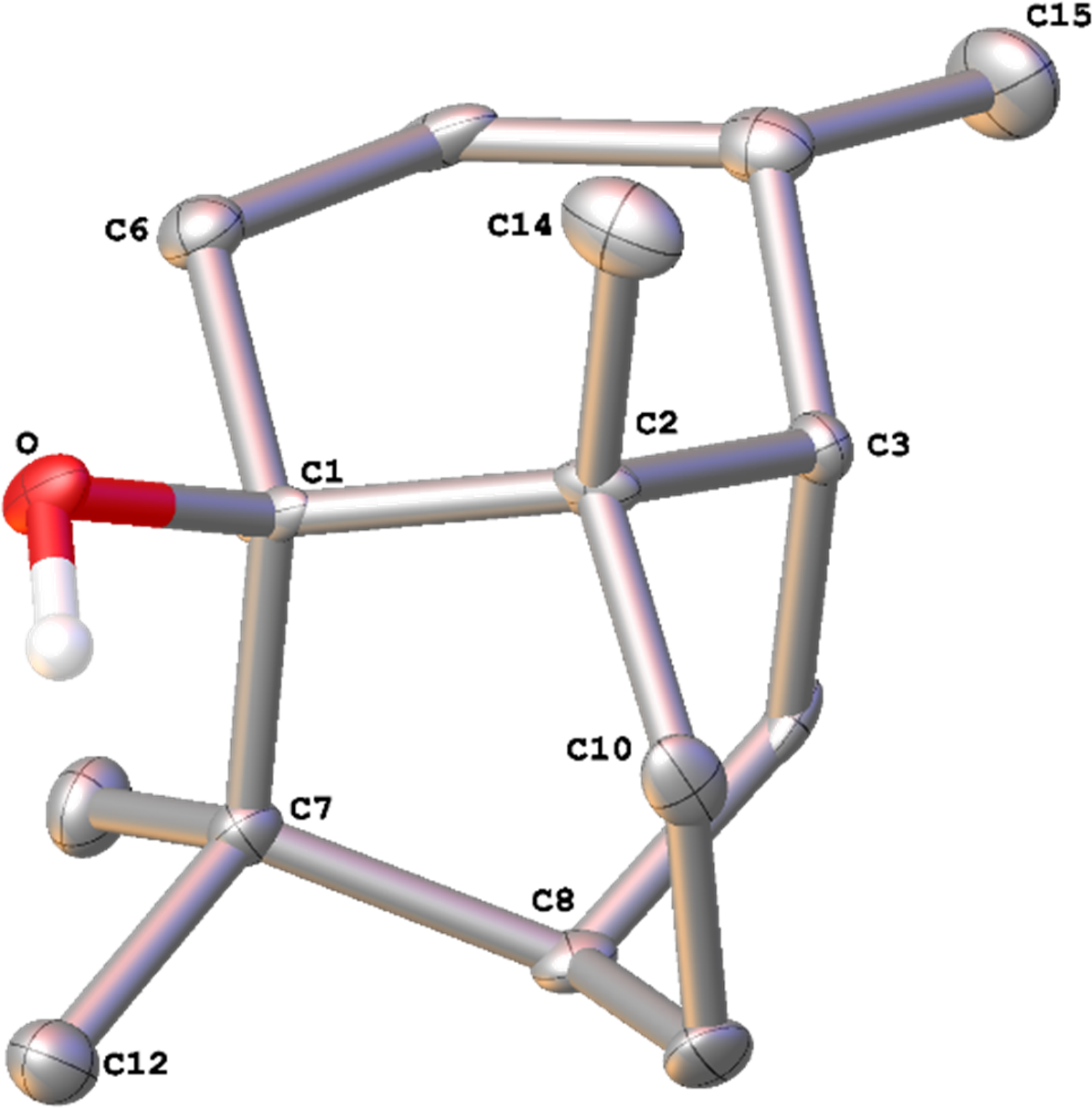
ORTEP representation of patchoulol (**3**). Cu Kα, Flack parameter: −0.1(2); P2(true) = 1.000, P3(false) = 0.6·10^−5^).

In order to reinvestigate the biosynthesis of patchoulol (**3**) the synthetic gene for patchoulol synthase from *P. cablin* was cloned and expressed in *Escherichia coli* [8]. The purified protein (Figure S1 in Supporting Information File 1) converted FPP into **3** as the main product, besides several side products (see Figure S2 in Supporting Information File 1). A reference sample of **3** was isolated from patchouli oil and its structure was confirmed by NMR spectroscopy (Supporting Information File 1, Table S1 and Figures S3–S10). So far, only the X-ray structure of the chromate diester [6] and a Mo Kα structure of **3** were reported (CCDC no. 1491695) [17], but these data did not allow to conclude on the absolute configuration of compound **3**. We now obtained **3** as a crystalline material and performed an X-ray structural analysis through anomalous dispersion using Cu Kα irradiation (Table S2 in Supporting Information File 1), resulting in the structure of **3** with the absolute configuration as shown in Figure 2.

**Scheme 4:**
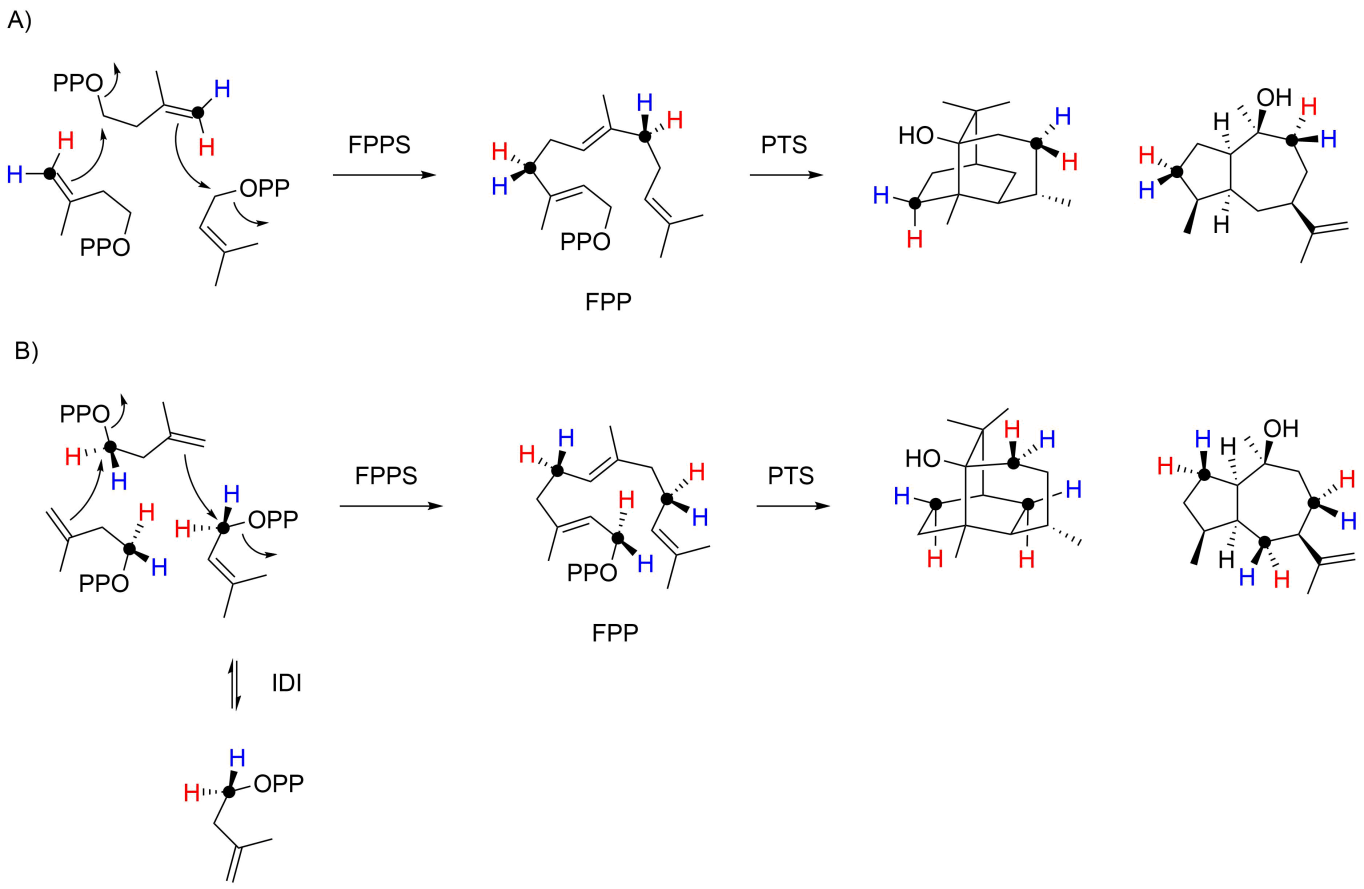
Determination of the absolute configurations of compounds **3** and **12** through stereoselective labelling experiments using (*E*)-(4-^13^C,4-^2^H)IPP (blue H = ^2^H) and (*Z*)-(4-^13^C,4-^2^H)IPP (red H = ^2^H), and (*R*)-(1-^13^C,1-^2^H)IPP (blue H = ^2^H) and (*S*)-(1-^13^C,1-^2^H)IPP (red H = ^2^H). Black dots indicate ^13^C-labelled carbons.

The absolute configuration of **3** was furthermore independently confirmed through a stereoselective deuteration strategy (Scheme 4; all labelling experiments of this study are summarised in Supporting Information File 1, Table S3). Using dimethylallyl diphosphate (DMAPP) and (*E*)- and (*Z*)-(4-^13^C,4-^2^H)isopentenyl diphosphate (IPP) [18] in conjunction with FPP synthase (FPPS) from *Streptomyces coelicolor* [19] and PTS (Supporting Information File 1, Figure S11), stereogenic centres of known configuration are introduced at the deuterated carbons. The NOESY-based assignment of the diastereotopic hydrogens at these carbons for the unlabelled compound then allows to conclude on the absolute configuration of alcohol **3**. A second set of experiments made use of (*R*)- and (*S*)-(1-^13^C,1-^2^H)IPP [20] that were enzymatically converted with isopentenyl diphosphate isomerase (IDI) from *E. coli* [20–21], FPPS, and PTS (Figure S12 in Supporting Information File 1). The additional ^13^C-labellings in these experiments serve for a sensitive monitoring of deuterium incorporation through HSQC spectroscopy. All X-ray and labelling experiments confirmed the absolute configuration of **3** as reported previously.

It is reasonable to assume that pogostol (**12**) as a side product of PTS has the absolute configuration as shown in Figure 1, but surprisingly its absolute configuration has never been formally established. Moreover, pogostol registered under the CAS number 21698-41-9 is even assigned the opposite absolute configuration as expected from these biosynthetic considerations. After a recent correction [22] of its initially reported relative configuration [23] that was shown to be erroneous by total synthesis [24], we now address the problem of the absolute configuration of **12** experimentally. For this purpose, compound **12** was re-isolated from patchouli oil and its NMR data were fully assigned (see Supporting Information File 1, Table S4 and Figures S13–S20). Since **12** is also a side product of PTS, the data obtained from the above described labelling experiments were then used to determine the absolute configuration of pogostol as (1*R*,4*S*,5*S*,7*R*,10*S*)-**12** (Scheme 4, and Figures S21 and S22 in Supporting Information File 1).

### Investigations on patchoulol biosynthesis by labelling experiments

The cyclisation mechanism from FPP to patchoulol (**3**) was investigated in isotopic labelling experiments. Our aim was not only to obtain results that can support one of the three mechanisms under discussion in the literature, but because of the partially contradictory findings also to perform experiments that may disprove some of the proposed mechanisms, in order to obtain a refined understanding of the biosynthesis of compound **3**.

**Scheme 5:**
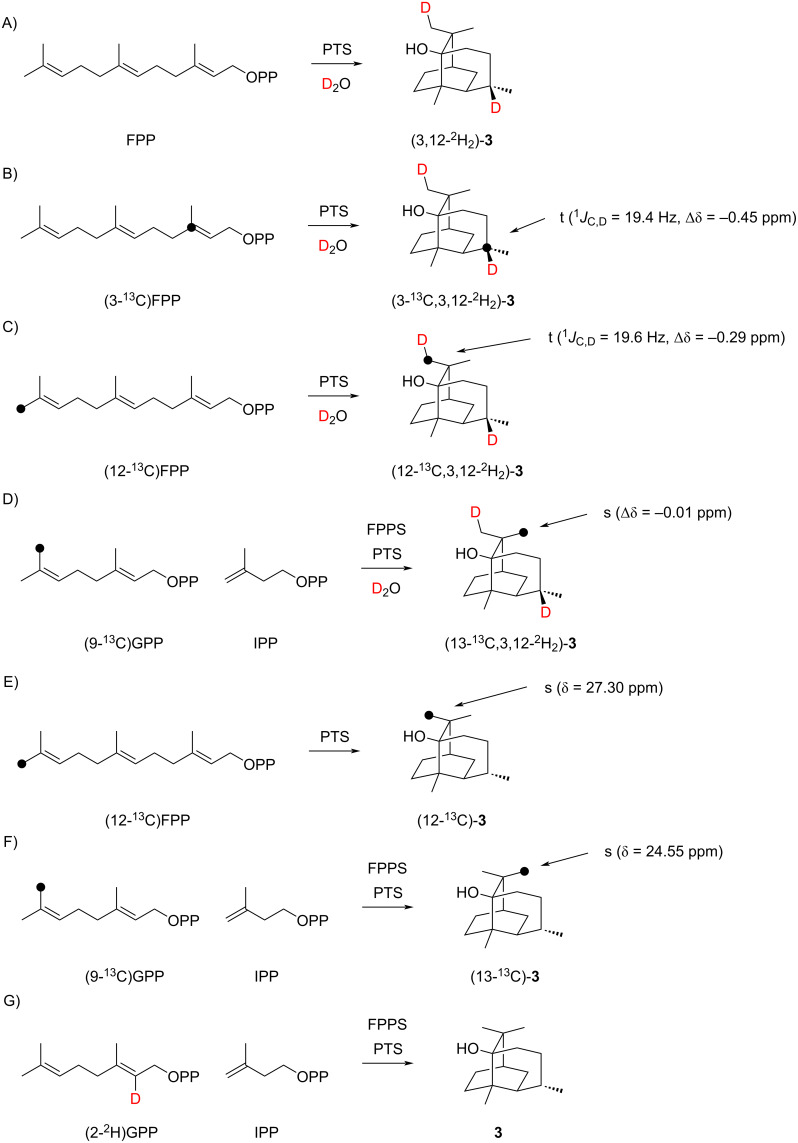
Labelling experiments on the biosynthesis of patchoulol (**3**, part 1). Black dots indicate ^13^C-labelled carbons.

In a first experiment, repeating earlier findings by Ekramzadeh et al. [13], the uptake of deuterium during an incubation of FPP with PTS in deuterium oxide buffer was investigated, revealing incorporation of two deuterium atoms into **3** (Scheme 5A and Figure S23 in Supporting Information File 1). This result is in agreement with the mechanism proposed by Akhila et al. (Scheme 2) [10], but not with the alternative mechanisms of Scheme 1 and Scheme 3, and therefore the next experiments focussed on gaining further evidence for the mechanism of Scheme 2. The site of incorporation for the deuterium uptake was evident from incubations of (3-^13^C)FPP and (12-^13^C)FPP [25] in deuterium oxide (Scheme 5B and 5C). The ^13^C NMR analysis of the obtained products showed slightly upfield-shifted triplets for C3 (Δδ = −0.45 ppm, *J* = 19.4 Hz) and C12 (Δδ = −0.29 ppm, *J* = 19.6 Hz) as a result of ^1^*J*_C,D_ couplings (see Figure S24B and S24C in Supporting Information File 1), again in full agreement with the mechanism by Akhila et al. [10]. A control experiment with (13-^13^C)FPP, enzymatically prepared from (9-^13^C)GPP [26], and IPP with FPPS, resulted in a singlet with a very small upfield shift (Δδ = −0.01 ppm) in the ^13^C NMR (Scheme 5D and Figure S24D in Supporting Information File 1), indicating a deuterium incorporation two positions away from C13 and a clear stereochemical course for the geminal methyl groups C12 and C13 of FPP. This was also confirmed through ^13^C-labelling experiments with (12-^13^C)FPP and (9-^13^C)GPP plus IPP in non-deuterated aqueous environment (Scheme 5E and 5F and Figure S24E and S24F in Supporting Information File 1). These experiments together with a detailed inspection of the NOESY spectrum of **3** also indicated that the assigned sites of incorporation of labellings from C12 of FPP by Akhila et al. (Scheme 2B) and by Ekramzadeh et al. (Scheme 2C) must be corrected, i.e., the carbons in **3** derived from the geminal Me groups C12 and C13 of FPP must be exchanged. Finally, the mechanism proposed by Akhila et al. includes a deprotonation step from C6 of FPP towards the neutral intermediate **6**. The enzymatic conversion of (2-^2^H)GPP [26] and IPP with FPPS into (6-^2^H)FPP and its subsequent cyclisation with PTS resulted in the formation of non-labelled **3**, in agreement with this deprotonation step (Scheme 5G and Figure S25 in Supporting Information File 1).

**Scheme 6:**
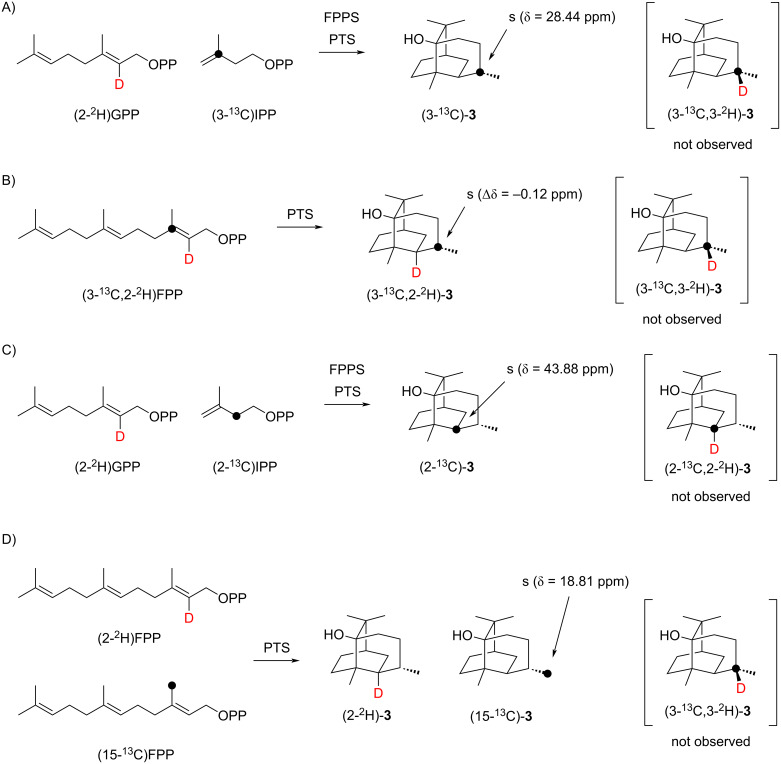
Labelling experiments on the biosynthesis of patchoulol (**3**, part 2). Black dots indicate ^13^C-labelled carbons.

Notably, not only the double deuterium uptake into **3** from D_2_O, but also the loss of deuterium from (6-^2^H)FPP contradicts the mechanisms of Scheme 1 and Scheme 3 that both propose a migration of hydrogen from C6 to C3, either through a 1,4- or a 1,3-hydride shift. An additional experiment with (2-^2^H)GPP, (3-^13^C)IPP [27], FPPS, and PTS produced a clear singlet in the ^13^C NMR spectrum for C3 of compound **3** (Scheme 6A and Figure S26 in Supporting Information File 1). Thus, there is no evidence, also not for a minor participation, for the proposed 1,4- or 1,3-hydride shifts of Scheme 1 and Scheme 3. Despite their proposal of a 1,3-hydride transfer for the conversion of **H** to **J** (Scheme 3), Faraldos et al. have pointed out that instead two sequential 1,2-hydride migrations through **I** would be easier to understand [14]. To investigate whether such alternative 1,2-hydride shifts take part, incubation experiments were performed with (3-^13^C,2-^2^H)FPP [28] plus PTS, and with (2-^2^H)GPP and (2-^13^C)IPP [27] plus FPPS and PTS (Scheme 6B and 6C), but in both cases the product analysis by ^13^C NMR spectroscopy showed only singlet signals for C3 and C2 of **3**, respectively (in the first case associated with a small upfield shift of Δδ = −0.12 ppm as a result of deuterium in the neighbouring position to C3, Figure S27 in Supporting Information File 1). Moreover, no triplet signals indicative for a direct ^13^C-^2^H bond were observed, ruling out the participation of two sequential 1,2-hydride shifts in the **H** to **J** transformation. To re-investigate the suggested intermolecular proton exchange in the biosynthesis of **3** (Scheme 3C) [14], an incubation experiment with the mixed substrates (2-^2^H)FPP [27] and (15-^13^C)FPP [24] was performed (Scheme 6D). Their conversion with PTS only resulted in a singlet for labelled C15 of **3** in the ^13^C NMR spectrum, but no upfield-shifted triplet (Figure S28, Supporting Information File 1), demonstrating that the hypothetical intermolecular proton shift does not take place. Instead, an additional incorporation of deuterium into the substrate during synthesis, contaminating the target compound (2-^2^H)FPP with some (2,15-^2^H_2_)FPP, seems to be the more likely explanation for the deuteration of **3** at C15 observed by Faraldos et al. [14]. This can also much better explain the deuterium content observed by GC–MS in the PTS products that were proposed to transfer deuterium to **3**. Following the mechanism of Scheme 3C, compounds such as **5**, if indeed obtained from pure (2-^2^H)FPP, should not show any residual deuterium content, if they donate their deuterium to **3**. The analytical data in reference [14] in fact show that **5** does contain deuterium, only one deuterium atom less than in **3**, but this deuterium loss for **5** is best explained by the terminal deprotonation step from C6.

### Investigations on patchoulol biosynthesis by DFT calculations

**Figure 3:**
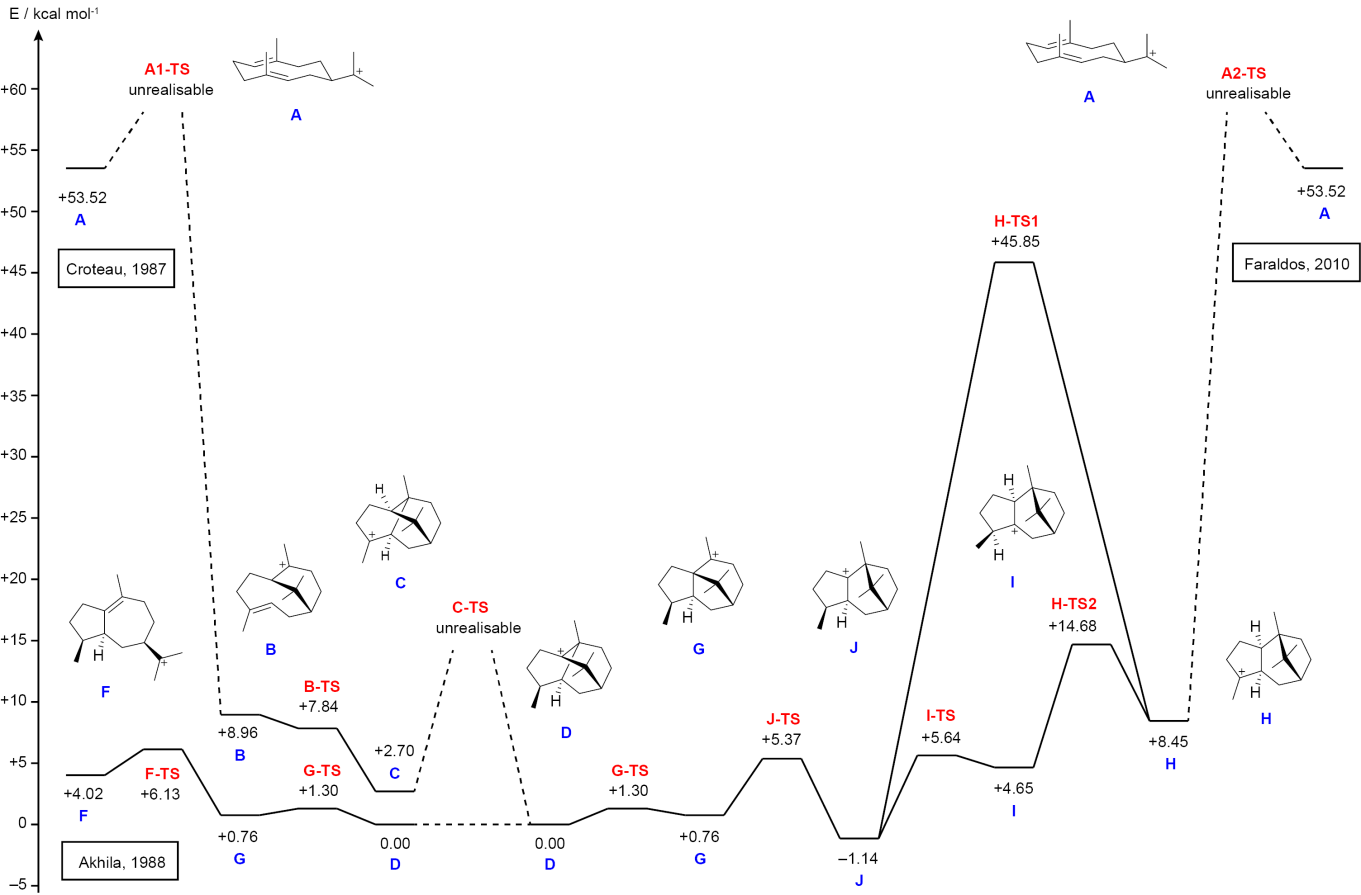
Energy profile from DFT calculations (Gibbs energies at 298 K, mPW1PW91/6-311 + G(d,p)//B97D3/6-31G(d,p)) for the three mechanisms of **3** biosynthesis by Croteau et al. [9], Akhila et al. [10], and Faraldos et al. [14]. The direct precursor of **3** in all three mechanisms, cation **D**, was set to 0.00 kcal/mol.

The biosynthesis of **3** was also investigated by DFT calculations (Figure 3). For the mechanism proposed by Croteau et al. (Scheme 1) [9], the cyclisation of the (*E*,*E*)-germacradienyl cation (**A**) to **B** could not be realised. The further reaction of **B** to **C** is barrierless, but the proposed 1,4-hydride shift to **D** is geometrically impossible and also cannot be realised by computations.

For the formation of the bicyclic cation **E** according to Akhila et al. (Scheme 2) [10] DFT calculations have been performed previously by us as part of a general study on guaiane sesquiterpenes from germacrene A (**8**) [29]. After reprotonation of the neutral intermediate **6** to **F** the next cyclisation to **G** and Wagner–Meerwein rearrangement to **D** can be realised with low TS barriers.

Also for the cyclisation of **A** to **H** as suggested by Faraldos et al. [14] the DFT calculations showed a strong steric repulsion that cannot be realised computationally. The 1,3-hydride shift from **H** to **J** is associated with a very high TS barrier (37.4 kcal/mol), while the sequence of two 1,2-hydride migrations via **I** to **J** would indeed be much easier. The final transformations involving two Wagner–Meerwein rearrangements through **G** and **D** can proceed smoothly.

### Isolation of guaia-1,11-dien-1-ol from patchouli oil

**Figure 4:**
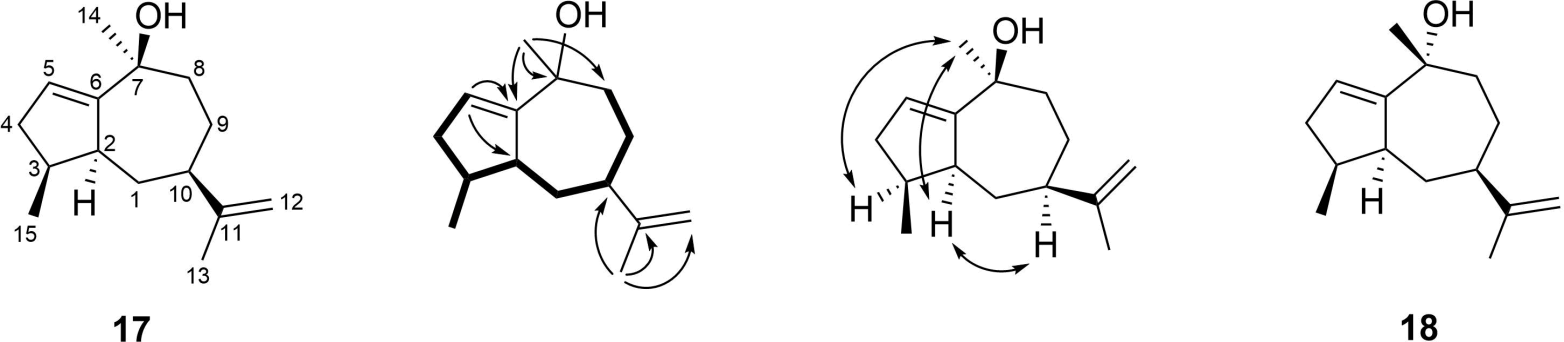
Structure elucidation of (2*S*,3*S*,7*S*,10*R*)-guaia-1,11-dien-10-ol (**17**) and structure of its known stereoisomer (2*S*,3*S*,7*R*,10*R*)-guaia-1,11-dien-10-ol (**18**). Bold lines indicate ^1^H,^1^H-COSY correlations, single-headed arrows indicate key HMBC correlations and double-headed arrows key NOESY correlations.

Fractionation of patchouli oil by column chromatography resulted in the isolation of the new natural product **17** [HRMS–ESI (*m*/*z*): 221.1904 [M + H]^+^, calculated for C_15_H_25_O^+^ 221.1900 and [α]_D_^25^ = −7.7, (*c* 0.26, benzene)] whose structure was elucidated by NMR spectroscopy (Table 1 and Figures S29–S35 in Supporting Information File 1). The ^13^C NMR spectrum showed signals for 15 carbons, including three Me groups, four olefinic carbons (two quarternary, one CH and one CH_2_), and a tertiary alcohol, suggesting the structure of an oxidised (dehydrogenated) bicyclic sesquiterpene alcohol (Figure 4). The ^1^H,^1^H-COSY spectrum revealed one large contiguous spin system C-2-3-4(15)-5-6-7-8-9. HMBC correlations from H_3_-13 to C-7, C-11, and C-12 indicated an isopropenyl group attached to C-7, while additional HMBC correlations from H_3_-14 to C-8, C-9, and C-10 and from H-2 to C-3, C-5, and C-10 completed the planar structure of **17**. Key NOESY correlations from H-4 and H-5 to H_3_-14 and from H-5 to H-7 placed these groups on one hemisphere of the molecule, revealing the structure of guaia-1,11-dien-10-ol. Based on the very likely biosynthetic relationship to the products of patchoulol synthase (especially **12**, Figure 1), the absolute configuration was tentatively assigned as (4*S*,5*S*,7*R*,10*S*)-**17**. A stereoisomer of this compound, (4*S*,5*S*,7*R*,10*R*)-**18**, was reported before from *Hyptis suaveolens* [30] and has been obtained by synthesis from α-bulnesene (**6**) with an optical rotation of [α]_D_^29^ = −79.2 (*c* 0.25, CHCl_3_) [31–32]. The negative optical rotation of **17** ([α]_D_^25^ = −7.7 (*c* 0.26, C_6_D_6_)) in comparison to the negative optical rotation of **18** further supports the tentatively assigned absolute configuration for **17**.

**Table 1:** NMR data of compound **17** (700 MHz, C_6_D_6_).

C^a^	type	^13^C	^1^H

1	CH_2_	35.62	1.75 (m, H_α_) 1.13 (ddd, 12.4, 12.4, 12.4, H_β_)
2	CH	47.84	2.06 (dddd, *J* = 12.3, 7.3, 2.1, 2.1)
3	CH	40.02	2.23 (dddq, *J* = 10.1, 7.2, 7.2, 7.2)
4	CH_2_	38.13	2.15 (ddd, *J* = 15.6, 7.7, 3.0, H_α_) 1.89 (dddd, *J* = 15.6, 10.1, 1.8, 1.8, H_β_)
5	CH	124.12	5.69 (dd, *J* = 2.9, 1.8)
6	C_q_	159.17	–
7	C_q_	73.38	–
8	CH_2_	42.68	1.76 (m, H_β_) 1.47 (m, H_α_)
9	CH_2_	29.20	1.53 (m, H_α_) 1.46 (m, H_β_)
10	CH	50.80	1.87 (m)
11	C_q_	151.90	–
12	CH_2_	108.87	4.80 (m, H*_Z_*) 4.74 (m, H*_E_*)
13	CH_3_	20.70	1.65 (dd, *J* = 1.3, 0.8)
14	CH_3_	32.45	1.26 (s)
15	CH_3_	15.47	0.91 (d, *J* = 7.0)

^a^Carbon numbering as shown in Figure 4; ^b^multiplicities are indicated by s = singlet, d = doublet, q = quartet, m = multiplet; coupling constants *J* are given in hertz.

## Conclusion

Different contradictory mechanisms for patchoulol biosynthesis have been discussed in the literature. The present study resolves this situation through isotopic labelling experiments. These experiments support the passage of two neutral intermediates, germacrene A and α-bulnesene, that become reactivated by reprotonations, as shown by incubation experiments in deuterium oxide buffer. These observations are in line with the proposed mechanisms by Akhila et al. [10] and Ekramzadeh et al. [13], with minor corrections regarding the stereochemical course of the geminal Me groups of FPP. Because it is possible that multiple mechanisms operate simultaneously, we also performed experiments to exclude other proposals made by Croteau et al. [9] and Faraldos et al. [14]. For this purpose, ^13^C-labelled substrates were used in conjunction with deuterium labelling. These substrates have the advantage that the incorporation of labelling can be detected and localised through ^13^C NMR spectroscopy with very high sensitivity, but no hints for critical steps such as 1,3- or 1,4-hydride shifts or intermolecular deuterium transfers as suggested in these studies were obtained. The results from labelling experiments are furthermore fully supported by DFT calculations. Our computational work also demonstrated that the mechanisms by Croteau et al. [9] and Faraldos et al. [14] are difficult to understand, while the mechanism by Akhila et al. [10] can proceed via low transition state barriers. As discussed above, the mistake in the mechanistic work by Faraldos et al. [14] seems to reside in an impure starting material (2-^2^H)FPP containing additional deuterium at C15, but it is difficult to understand the results by Croteau et al. [9]. As a general comment we can only state, how difficult it was to perform the old work using radioactive labellings, especially in terms of localising the site of incorporation by chemical degradations. It should be emphasised how fascinating and how deep the insights of many of such studies are. Today ^13^C and ^2^H-labellings in conjunction with NMR and MS-based analysis can be used, with strong advantages over radioactive labellings, not only from a safety perspective, but also with respect to the ease of data interpretation. Overall, our study gives another example of terpene biosynthesis through neutral intermediates, and more specifically another example of sesquiterpene biosynthesis through the widespread biosynthetic intermediate germacrene A [11].

## Supporting Information

File 1 Experimental details, characterisation data and copies of spectra.
