# Supplementary material for: The enzyme mechanism of patchoulol synthase
Source: Beilstein J Org Chem. 2022 Jan 3;18:13–24. doi: 10.3762/bjoc.18.2 (PMC8744462; doi:10.3762/bjoc.18.2)
Supplement: File 1 — Experimental details, characterisation data and copies of spectra. [file Beilstein_J_Org_Chem-18-13-s001.pdf]

BEILSTEIN JOURNAL OF ORGANIC CHEMISTRY

Supporting Information

for

The enzyme mechanism of patchoulol synthase

Houchao Xu, Bernd Goldfuss, Gregor Schnakenburg and Jeroen S. Dickschat

Beilstein J. Org. Chem. **2022,** *18,* 13–24. doi:10.3762/bjoc.18.2

Experimental details, characterisation data and copies of spectra

License and Terms: This is a supporting information file under the terms of the Creative Commons Attribution License (https://creativecommons.org/licenses/by/4.0). Please note that the reuse, redistribution and reproduction in particular requires that the author(s) and source are credited and that individual graphics may be subject to special legal provisions.
The license is subject to the *Beilstein Journal of Organic Chemistry* terms and conditions: (https://www.beilstein-journals.org/bjoc/terms)

Isolation of patchoulol (3), pogostol (12) and (2*S*,3*S*,7*S*,10*R*)-guaia-1,11-dien-10-ol (17) from patchouli oil

Patchouli oil (10 g) was subjected to the silica gel column chromatography. Elution with a pentane/Et_2O gradient (1:0, 4:1, and then 3:1) yielded 17 fractions. Fr. 10 (2.5 g) was identified as pure **3**. Fr. 11 (0.3 g) was chromatographed on silica gel using a mobile phase of pentane/Et_2O 4:1 to obtain fractions 11A–11C. Fr. 11A (26 mg) and Fr. 11C (0.2 g) were further purified by preparative HPLC on a chiral stationary phase to give **17** (4 mg) and **12** (0.12 g), respectively.

Patchoulol (3). Yield: 2.5 g (from 10 g patchouli oil, 25%). TLC (pentane / diethyl ether = 1:1): R_f = 0.62. GC (HP5-MS): *I* = 1690. IR (diamond ATR): $\tilde{v}$ / cm^{-1} = 2934 (s), 2870 (s), 1467 (m), 1457 (m), 1376 (w), 1056 (w), 1040 (m), 1000 (w), 982 (w). HR-MS (ESI^+): calc. for $[C_{15}H_{25}]^+$ *m*/*z* = 205.1951; found: *m*/*z* = 205.1950. Optical rotary power: $[\alpha]_D^{25}$ = –102.8 (*c* 0.21, benzene). NMR data are given in Table S1.

Pogostol (12). Yield: 120 mg (from 10 g patchouli oil, 1.2%). TLC (pentane / diethyl ether = 1:1): R_f = 0.52. GC (HP5-MS): *I* = 1681. IR (diamond ATR): $\tilde{v}$ / cm^{-1} = 2947 (s), 2928 (s), 2870 (m), 1644 (w), 1454 (w), 1375 (w), 1103 (w), 1066 (w), 885 (m), 543 (w). HR-MS (ESI^+): calc. for $[C_{15}H_{27}O]^+$ *m*/*z* = 223.2056; found: *m*/*z* = 223.2058. Optical rotary power: $[\alpha]_D^{25}$ = –27.0 (*c* 0.10, benzene). NMR data are given in Table S4.

(2*S*,3*S*,7*S*,10*R*)-Guaia-1,11-dien-10-ol (17). Yield: 4 mg (from 10 g patchouli oil, 0.04%). TLC (pentane / diethyl ether = 1:1): R_f = 0.57. GC (HP5-MS): *I* = 1610. IR (diamond ATR): $\tilde{v}$ / cm^{-1} = 2960 (m), 2925 (s), 2871 (m), 1643 (w), 1448 (m), 1374 (w), 1028 (m), 886 (m), 812 (w), 670 (w). HR-MS (ESI^+): calc. for $[C_{15}H_{27}O]^+$ *m*/*z* = 221.1900; found: *m*/*z* = 221.1904. Optical rotary power: $[\alpha]_D^{25}$ = –7.7 (*c* 0.26, benzene). NMR data are given in Table 1 of main text.

HPLC

Analytical scale HPLC separation was carried out using a PLATINblue series HPLC system (Knauer, Berlin, Germany), equipped with a PAD-1 photodiode array detector (190–1000 nm) and a KNAUER Eurospher II 100-3 C18 column (3.0 μm; 2.0 mm × 100 mm). The UV–vis absorption was monitored at 190–600 nm.

Preparative scale HPLC purification was performed on an Azura series HPLC system (Knauer, Berlin, Germany) with a multiwavelength detector MWL 2.1L (190–700 nm) using a KNAUER Eurospher II 100-5 C18P column (5 μm, 250 × 16 mm).

GC–MS

GC–MS analyses were carried out on a 7890B/5977A series gas chromatography–mass selective detector (Agilent, Santa Clara, CA, USA). The GC was equipped with an HP5-MS fused silica capillary column (30 m, 0.25 mm i.d., 0.50 μm film; Agilent) and operated using the settings 1) inlet pressure: 77.1 kPa, He at 23.3 mL min^{-1}, 2) injection volume: 1–2 μL, 3) temperature program: 5 min at 50 °C then increasing 5 °C min^{-1} to 320 °C, 4) 60 s valve time, and 5) carrier gas: He at 1.2 mL min^{-1}. The MS was operated with settings 1) source: 230 °C, 2) transfer line: 250 °C, 3) quadrupole: 150 °C, and 4) electron energy: 70 eV.

NMR spectroscopy

NMR spectra were recorded at 298 K on a Bruker (Billerica, MA, USA) Avance III HD Cryo (700 MHz) NMR spectrometer. Spectra were measured in C_6D_6 and referenced against

solvent signals (^{1}H NMR, residual proton signal: δ = 7.16 ppm; ^{13}C NMR: δ = 128.06 ppm).[1]

IR spectroscopy
IR spectra were recorded on a Bruker α infrared spectrometer with a diamond ATR probehead. Peak intensities are given as s (strong), m (medium), w (weak), and br (broad).

Optical rotations
Optical rotations were recorded on a Modular Compact Polarimeter MCP 100 (Anton Paar, Graz, Austria). The temperature setting was 25 °C; the wavelength of the light used was 589 nm (sodium D line); the path-length was 10 cm, the compound concentrations *c* are given in g 100 mL^{-1}.

Gene expression and purification of PTS
A preculture of the *E. coli* transformants harbouring the plasmid pYE-AAS86323 was grown overnight at 37 °C in LB medium containing kanamycin (50 μg mL^{-1}). The expression cultures were then inoculated using 20 mL L^{-1} of preculture, followed by culturing at 37 °C with shaking in TB medium (24.0 g L^{-1} yeast extract, 20.0 g L^{-1} tryptone, 4.0 mL L^{-1} glycerol, 2.44 g L^{-1}, KH_2PO_4, 12.5 g L^{-1} K_2HPO_4) until an OD_{600} = 0.4 was reached. The cultures were cooled to 18 °C and protein expression was induced by addition of IPTG (400 mM in water, 1 mL L^{-1}). The expression cultures were shaken overnight at 18 °C and then centrifuged at 3.600*g* (4 °C). The supernatant was discarded and the cell pellet was resuspended in binding buffer (10 mL L^{-1} culture; 20 mM Na_2HPO_4, 500 mM NaCl, 20 mM imidazole, 1 mM $MgCl_2$, pH 7.4, 4 °C). The resulting suspension was subjected to ultra-sonication for cell lysis. The cell debris was removed by centrifugation (14.610*g*, 15 min, 4 °C) and the supernatant was loaded onto a Ni^{2+}-NTA affinity chromatography column (Super Ni-NTA, Generon, Slough, UK). The column was washed with binding buffer (2 × 10 mL L^{-1} culture), and the desired His-tagged protein was eluted using elution buffer (10 mL L^{-1} culture, 20 mM Na_2HPO_4, 500 mM NaCl, 500 mM imidazole, 1 mM $MgCl_2$, pH 7.4, 4 °C). Fractions containing protein were pooled, analysed by Bradford assay to determine the protein concentration (6 mg mL^{-1}) and by SDS-PAGE (Figure S1), and used for incubation experiments.

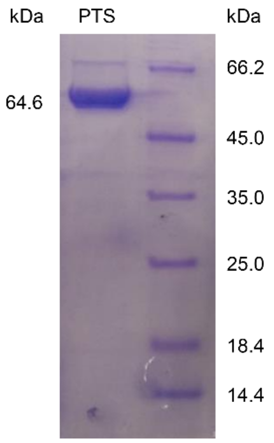

Figure S1: SDS-PAGE analysis of purified recombinant PTS.

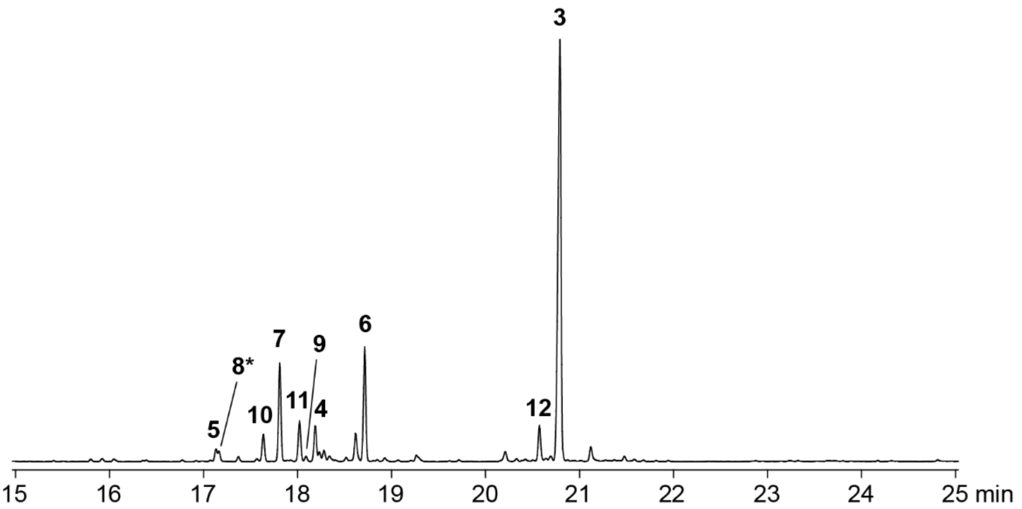

Figure S2: Total ion chromatogram of an extract from the incubation of FPP with PTS. Numbers at peaks refer to compound numbers in Figure 1 of main text. Germacrene A (**8**) was detected as its Cope rearrangement product β-elemene (**8***).

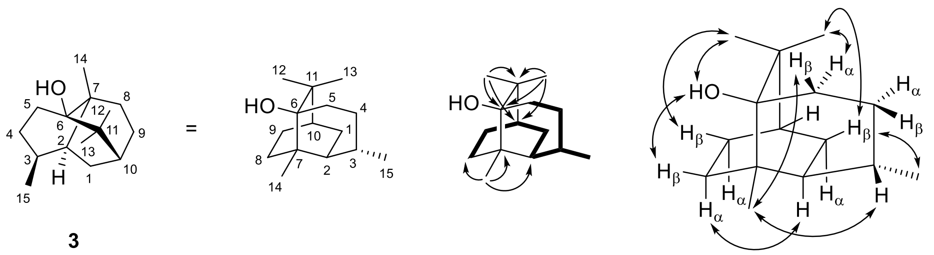

3

Figure S3: Structure elucidation of **3**. Bold: $^{1}H,^{1}H$-COSY, single-headed arrows: key HMBC, and double-headed arrows: key NOESY correlations.

Table S1: NMR data of patchoulol (**3**) in C_6D_6 recorded at 298 K.

C[a]	type	^{13}C[b]	^{1}H[b]
1	CH_2	24.95	1.37 (m, H_β)
			1.16 (m, H_α)
2	CH	43.88	1.28 (m)
3	CH	28.44	1.82 (m)
4	CH_2	28.96	1.27 (m, 2H)
5	CH_2	33.22	1.58 (dd, J = 13.6, 5.4, H_α)
			1.34 (m, H_β)
6	C_q	75.01	–
7	C_q	37.89	–
8	CH_2	29.18	1.92 (ddd, J = 13.6, 11.4, 7.6, H_β)
			0.96 (m, H_α)
9	CH_2	24.79	1.86 (m, H_β)
			1.16 (m, H_α)
10	CH	39.51	1.06 (m)
11	C_q	40.40	–
12	CH_3	27.30	1.09 (s)
13	CH_3	24.55	1.00 (s)
14	CH_3	20.89	0.80 (s)
15	CH_3	18.81	0.74 (d, J = 6.7)
	OH	–	0.57 (br s)

[a] Carbon numbering as shown in Figure S3. [b] Chemical shifts δ in ppm; multiplicity: s = singlet, d = doublet, m = multiplet, br = broad; coupling constants J are given in Hertz.

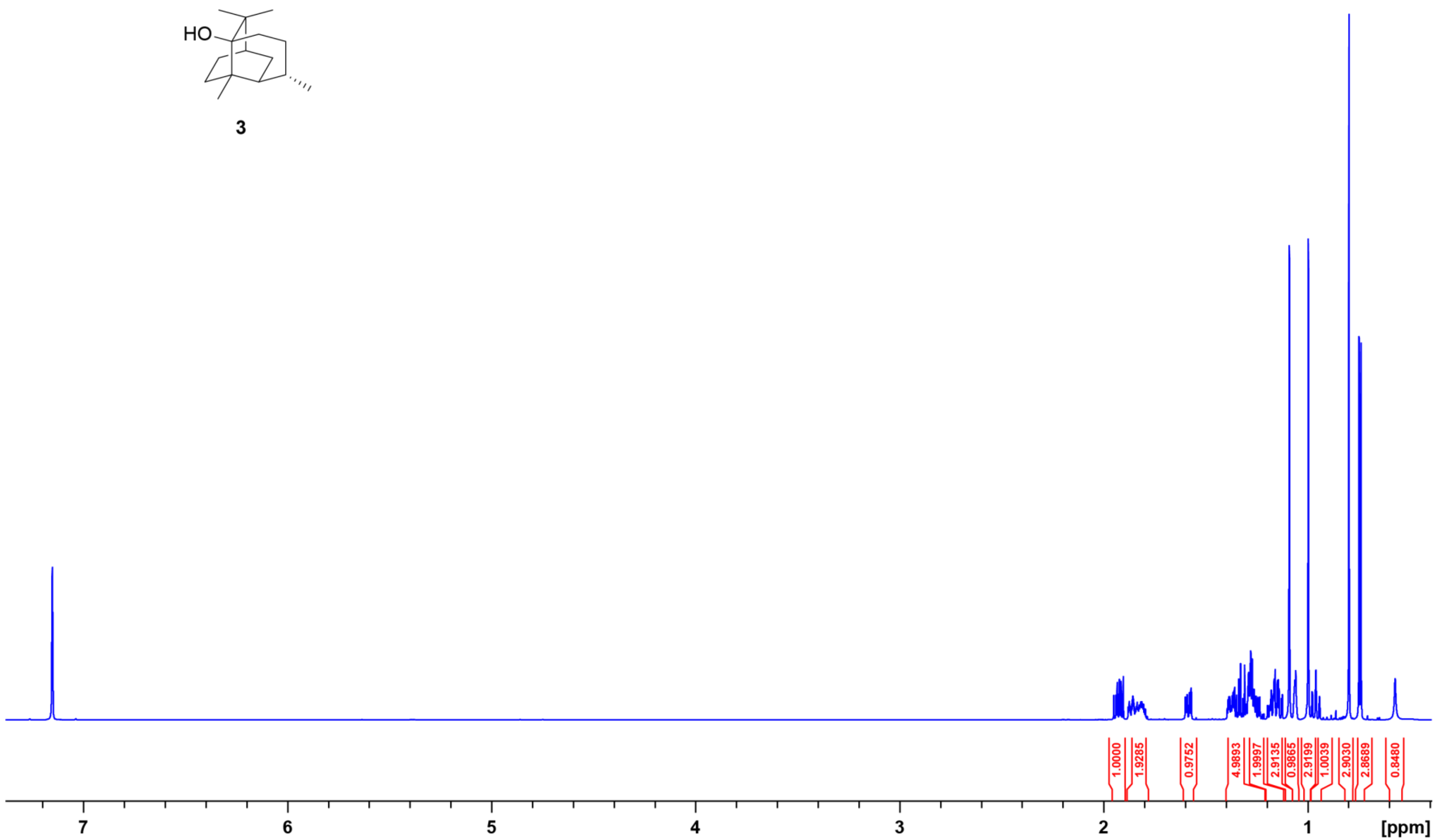

Figure S4: ^{1}H NMR spectrum of **3** (700 MHz, C_6D_6).

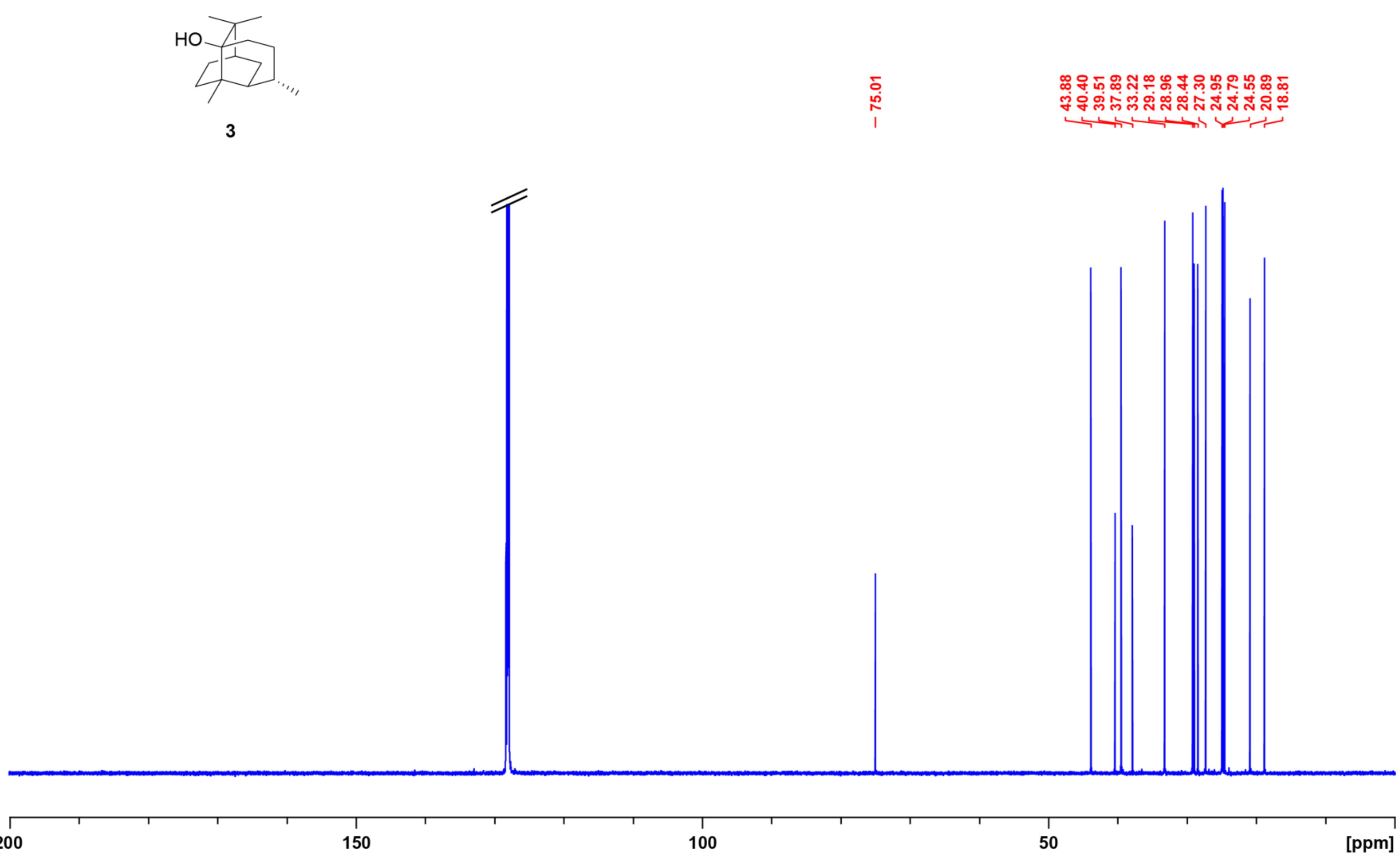

Figure S5: ^{13}C NMR spectrum of **3** (176 MHz, C_6D_6).

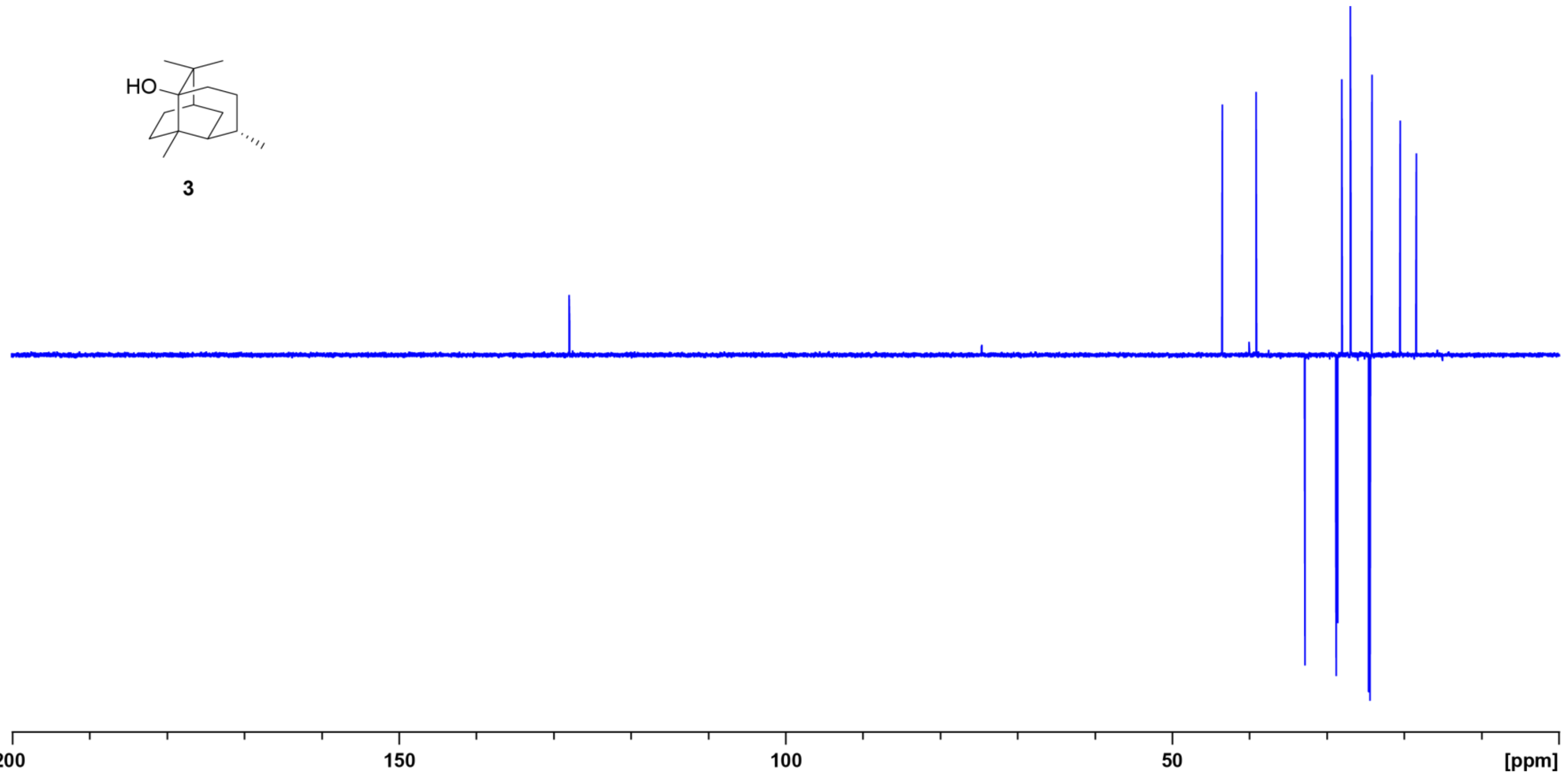

Figure S6: ^{13}C-DEPT spectrum of **3** (176 MHz, C_6D_6).

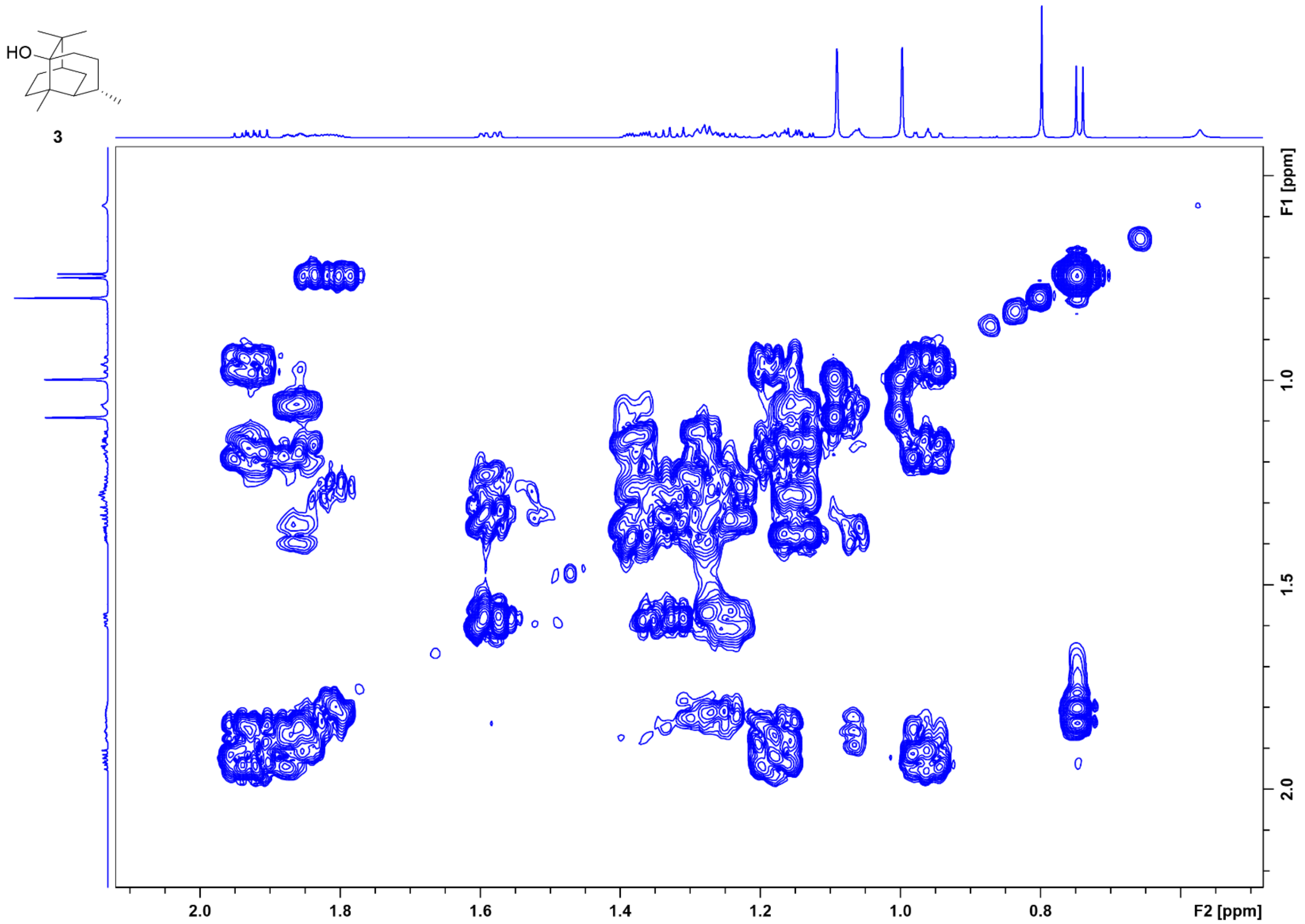

Figure S7: ^1H-^1H-COSY spectrum of **3** (700 MHz, C_6D_6).

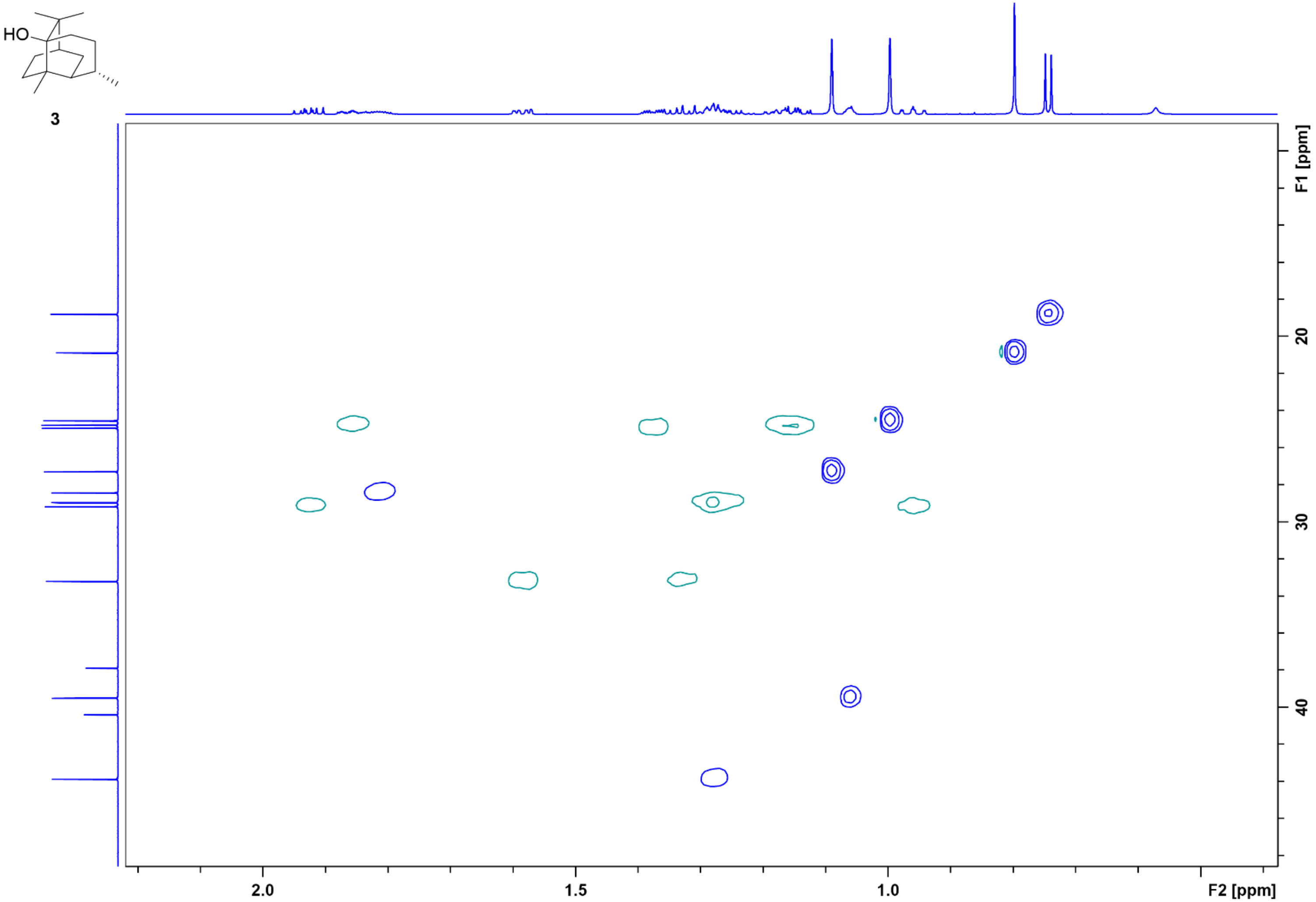

Figure S8: HSQC spectrum of **3** (C_6D_6).

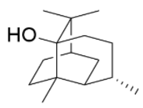

3

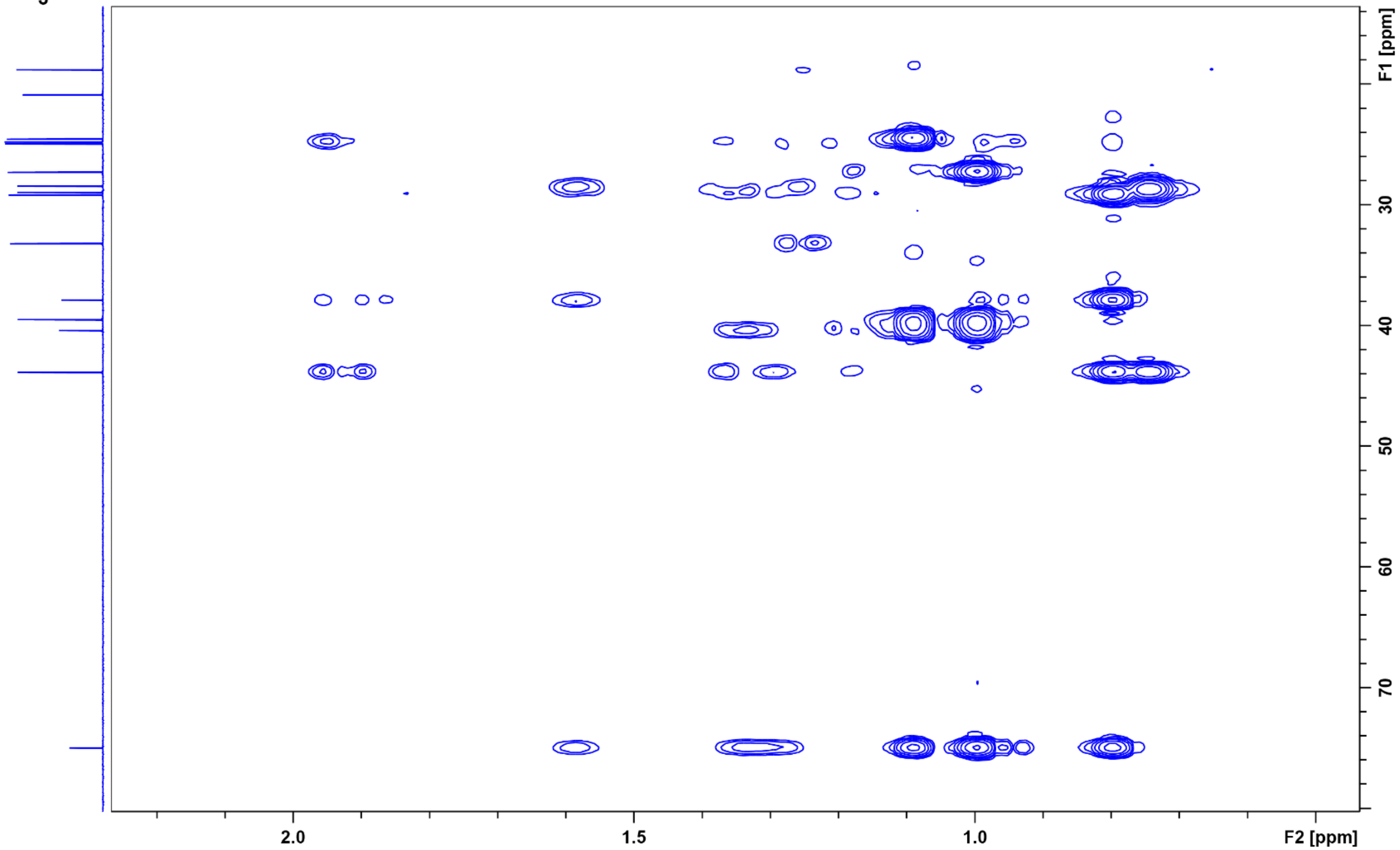

Figure S9: HMBC spectrum of **3** (C_6D_6).

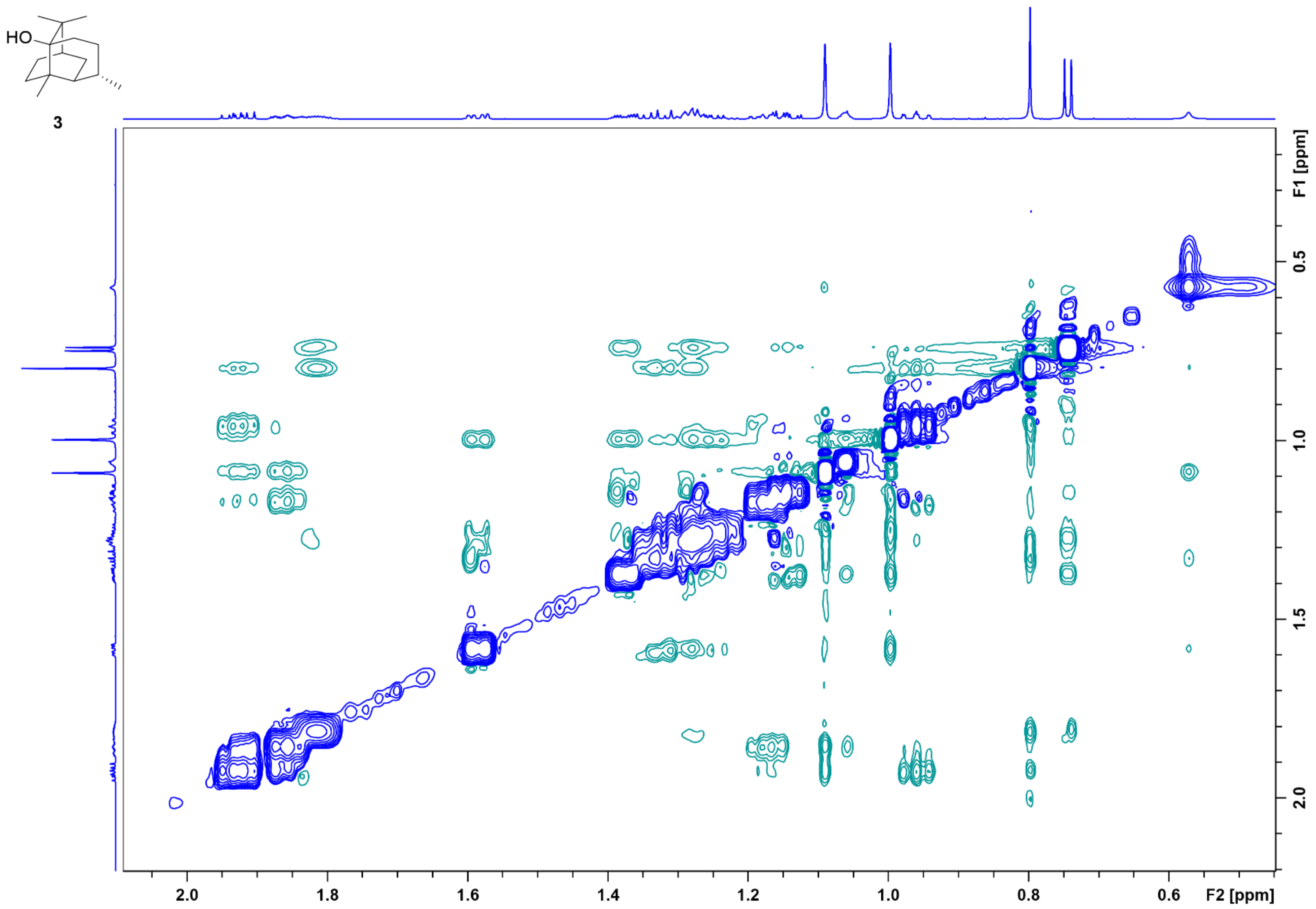

Figure S10: NOESY spectrum of **3** (C_6D_6).

Crystal structure determination of 3

Suitable colourless plate-like single crystals were grown by recrystallisation from pentane/diethyl ether 4:1 upon standing at −20 °C.

The data collection was performed on a Bruker D8-Venture diffractometer (area detector Photon I) using Cu Kα irradiation (λ = 1.54178 Å). The diffractometer was equipped with a low-temperature device (Oxford Cryostream 800er series, Oxford Cryosystems; set to 100K). Intensities were measured by fine-slicing ω and φ-scans and corrected for background, polarization, and Lorentz effects. A semi-empirical absorption correction was applied for the data set [2]. The structures were solved by direct methods and refined anisotropically by the least-square procedure implemented in the SHELX program system [3]. Hydrogen atoms were included using the riding model on the bound carbon atoms. The absolute configuration was estimated according to the Flack parameter of −0.1(2) and further investigated by determining quantitative estimates for the reliability of our assignment by using Bayesian statistics on Bijvoet differences by Hooft et al [4].

According to this the probability of the correct assignment of the absolute configuration assuming an enantiopure compound was determined to P2(true) = 1.000. The probability parameter of the correct assignment of the absolute configuration including the option of potential racemic twinning was determined to P3(true) = 0.960, whereas the probability of incorrect assignment is P3(false) = $0.6 \cdot 10^{-5}$.

Table S2: X-ray data of **3**.

crystal habitus	clear colourless plate
device type	Bruker D8 Venture
empirical formula	$C_{15}H_{26}O$
moiety formula	$C_{15}H_{26}O$
formula weight	222.36 g $mol^{–1}$
T / K	100.0
crystal system	hexagonal
space group	$P6_3$
a / Å	16.1658(6)
b / Å	16.1658(6)
c / Å	8.8956(4)
α / °	90
β / °	90
γ / °	120
V / $Å^3$	2013.26(17)
Z	6
$ρ_{calc}$ / g $cm^{–3}$	1.100
μ / mm^{-1}	0.498
F(000)	744.0
crystal size / mm^3	0.18 × 0.06 × 0.03
absorption correction	empirical
T_{min}; T_{max}	0.3194; 0.7535
radiation	CuKα (λ = 1.54178)
2Θ range for data collection / °	10.946 to 135.496°
completeness to Θ	0.989
index ranges	–18 ≤ h ≤ 17, –18 ≤ k ≤ 17, –10 ≤ l ≤ 10
reflections collected	9538
independent reflections	2356 [R_{int} = 0.0931, R_{sigma} = 0.0711]
data / restraints / parameters	2356 / 25 / 150
goodness-of-fit on F^2	1.117
final R indexes [I>=2σ (I)]	R_1 = 0.0611, wR_2 = 0.1385
final R indexes [all data]	R_1 = 0.0674, wR_2 = 0.1415
largest diff. peak/hole / e $Å^{-3}$	0.26/-0.26
Flack parameter	–0.1(2)
Hooft parameter(s)	0.0(2); P2(true) = 1.000 / P3(true) = 0.960 / P3(twin) = 0.04 / P3(false) = $0.6·10^{–5}$
CCDC deposition number	CCDC 2118370

Isotopic labelling experiments

Isotopic labelling experiments were performed with the substrates and enzymes as listed in Table S3. The reaction mixtures contained substrates (1 mg each) in aqueous NH_4HCO_3 solution (1 mL, 25 mM), enzyme elution fractions (2 mL each) and incubation buffer (5 mL, 50 mM Tris, 10 mM $MgCl_2$, 20 vol % glycerol, pH 8.2). After incubation with shaking at 28 °C overnight, the reaction mixtures were extracted with *n*-hexane (0.15 mL, entries 5 and 11) or C_6D_6 (0.6 mL + 0.2 mL, all other entries of Table S3). The extracts were dried with $MgSO_4$ and analysed by GC–MS and/or NMR.

Table S3: Labelling experiments with PTS.

entry	substrate	enzymes	results shown in
1	DMAPP + (*E*)-(4-^{13}C,4-^{2}H)IPP[5]	FPPS,[6] PTS	Figures S11 and S21
2	DMAPP + (*Z*)-(4-^{13}C,4-^{2}H)IPP[5]	FPPS, PTS	Figures S11 and S21
3	(*R*)-(1-^{13}C,1-^{2}H)IPP[7]	IDI,[7] FPPS, PTS	Figures S12 and S22
4	(*S*)-(1-^{13}C,1-^{2}H)IPP[7]	IDI, FPPS, PTS	Figures S12 and S22
5	FPP in $^{2}H_2O$	PTS	Figure S23
6	(3-^{13}C)FPP[8] in $^{2}H_2O$	PTS	Figure S24
7	(12-^{13}C)FPP[8] in $^{2}H_2O$	PTS	Figure S24
8	(9-^{13}C)GPP[9] + IPP in $^{2}H_2O$	FPPS, PTS	Figure S24
9	(12-^{13}C)FPP	PTS	Figure S24
10	(9-^{13}C)GPP + IPP	FPPS, PTS	Figure S24
11	(2-^{2}H)GPP[9] + IPP	FPPS, PTS	Figure S25
12	(2-^{2}H)GPP + (3-^{13}C)IPP[10]	FPPS, PTS	Figure S26
13	(3-^{13}C,2-^{2}H)FPP[11]	PTS	Figure S27
14	(2-^{2}H)GPP + (2-^{13}C)IPP[10]	FPPS, PTS	Figure S27
15	(2-^{2}H)FPP[10] + (15-^{13}C)FPP[8]	PTS	Figure S28

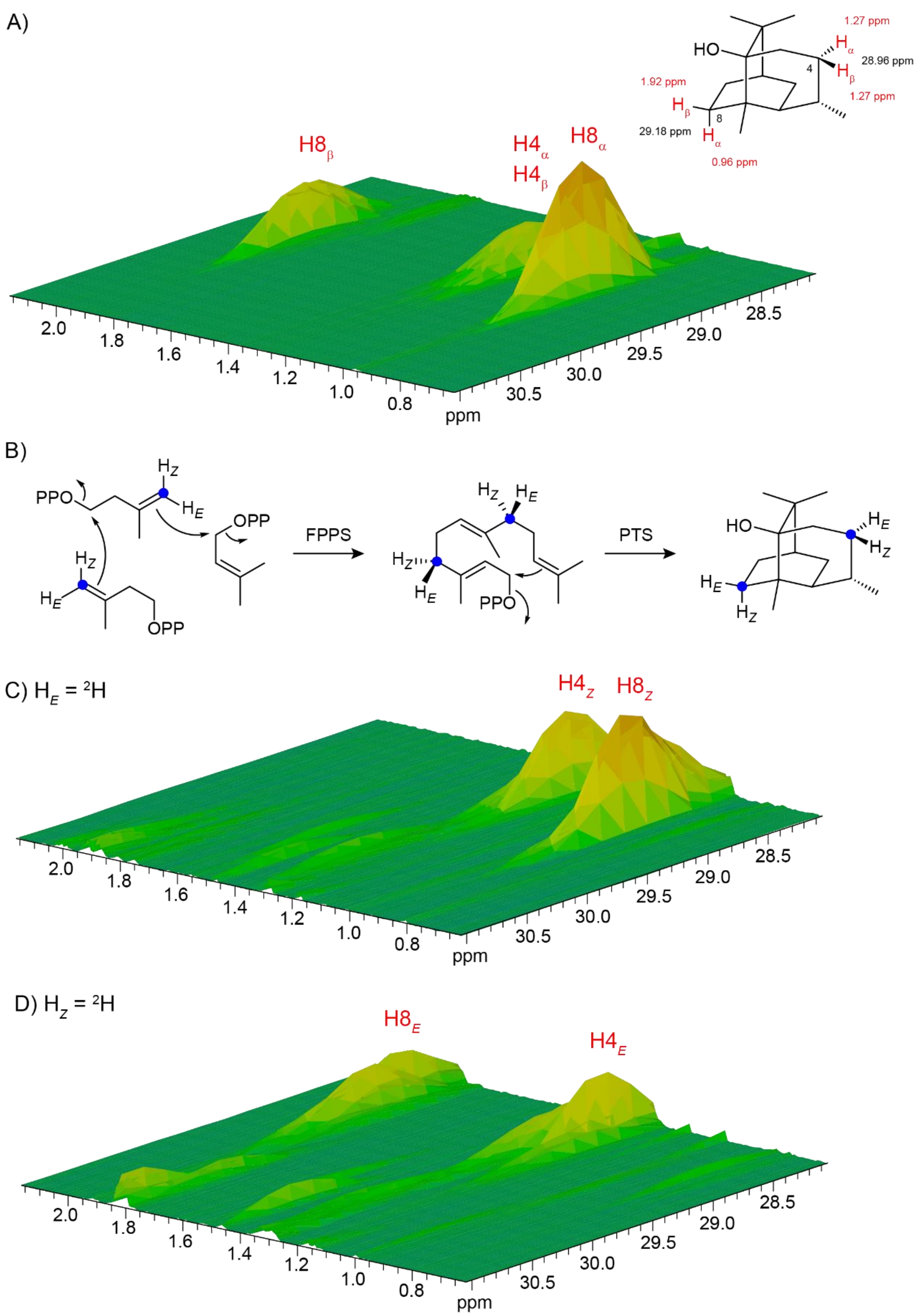

Figure S11: Determination of the absolute configuration of **3**. A) HSQC of unlabelled **3** showing crosspeaks for C4 and C8. HSQC of labelled **3** obtained from B) DMAPP and (*E*)-(4-^{13}C,4-^{2}H)IPP, and C) DMAPP and (*Z*)-(4-^{13}C,4-^{2}H)IPP with FPPS and PTS. The signals for deuterium incorporation are vanished, and because the configurations at deuterated carbons are known, the absolute configuration of **3** can be concluded.

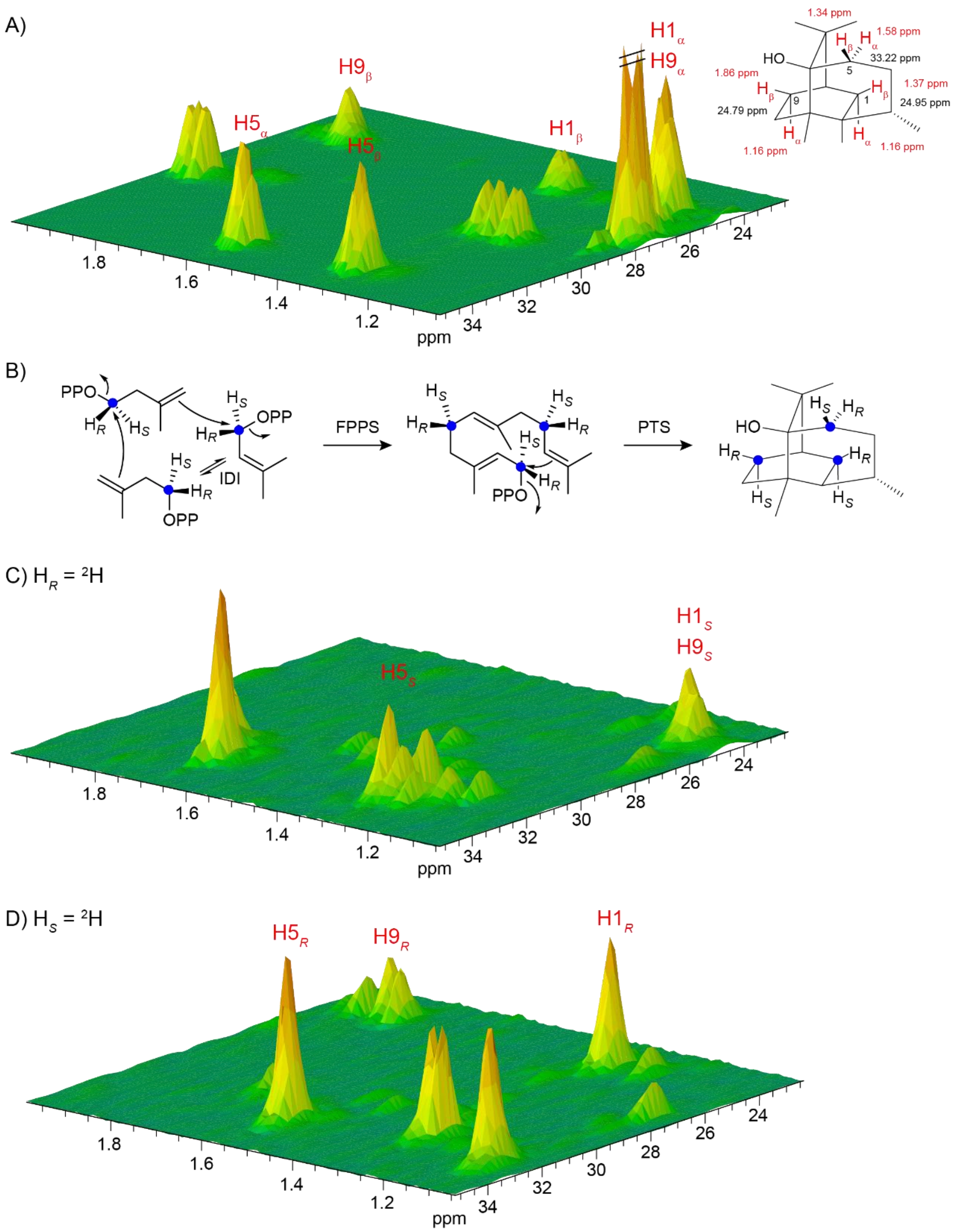

Figure S12: Determination of the absolute configuration of **3**. A) HSQC of unlabelled **3** showing crosspeaks for C1, C5 and C9. HSQC of labelled **3** obtained from B) (*R*)-(1-^{13}C,1-^{2}H)IPP, and C) (*S*)-(1-^{13}C,1-^{2}H)IPP with IDI, FPPS and PTS. The signals for deuterium incorporation are vanished, and because the configurations at deuterated carbons are known, the absolute configuration of **3** can be concluded.

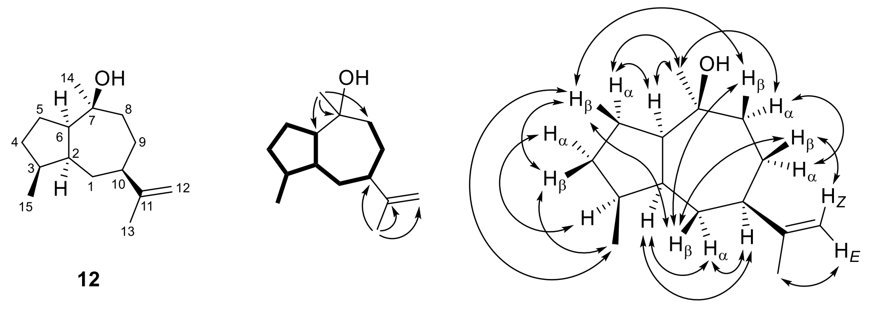

12

Figure S13: Structure elucidation of **12**. Bold: ^{1}H,^{1}H-COSY, single-headed arrows: key HMBC, and double-headed arrows: key NOESY correlations.

Table S4: NMR data of pogostol (**12**) in C_6D_6 recorded at 298 K.

C[a]	type	[13]C[b]	[1]H[b]
1	CH_2	28.64	1.45 (dm, *J* = 13.8, H_α)
			1.16 (m, H_β)
2	CH	46.24	1.91 (m)
3	CH	39.20	1.87 (m)
4	CH_2	31.35	1.59 (m, H_α)
			1.17 (m, H_β)
5	CH_2	26.39	1.55 (m, H_α)
			1.37 (m, H_β)
6	CH	55.90	1.90 (m)
7	C_q	74.05	–
8	CH_2	35.53	1.73 (m, H_β)
			1.36 (m, H_α)
9	CH_2	28.92	1.93 (m, H_α)
			1.39 (m, H_β)
10	CH	46.16	2.42 (m)
11	C_q	152.51	–
12	CH_2	108.28	4.83 (m, H_Z)
			4.72 (m, H_E)
13	CH_3	20.07	1.68 (dd, *J* = 1.4, 0.8)
14	CH_3	30.57	1.02 (s)
15	CH_3	16.36	0.85 (d, *J* = 6.9)
	OH	–	0.80 (br s)

[a] Carbon numbering as shown in Figure S13. [b] Chemical shifts δ in ppm; multiplicity: s = singlet, d = doublet, m = multiplet, br = broad; coupling constants *J* are given in Hertz.

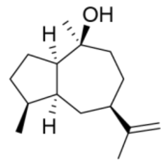

12

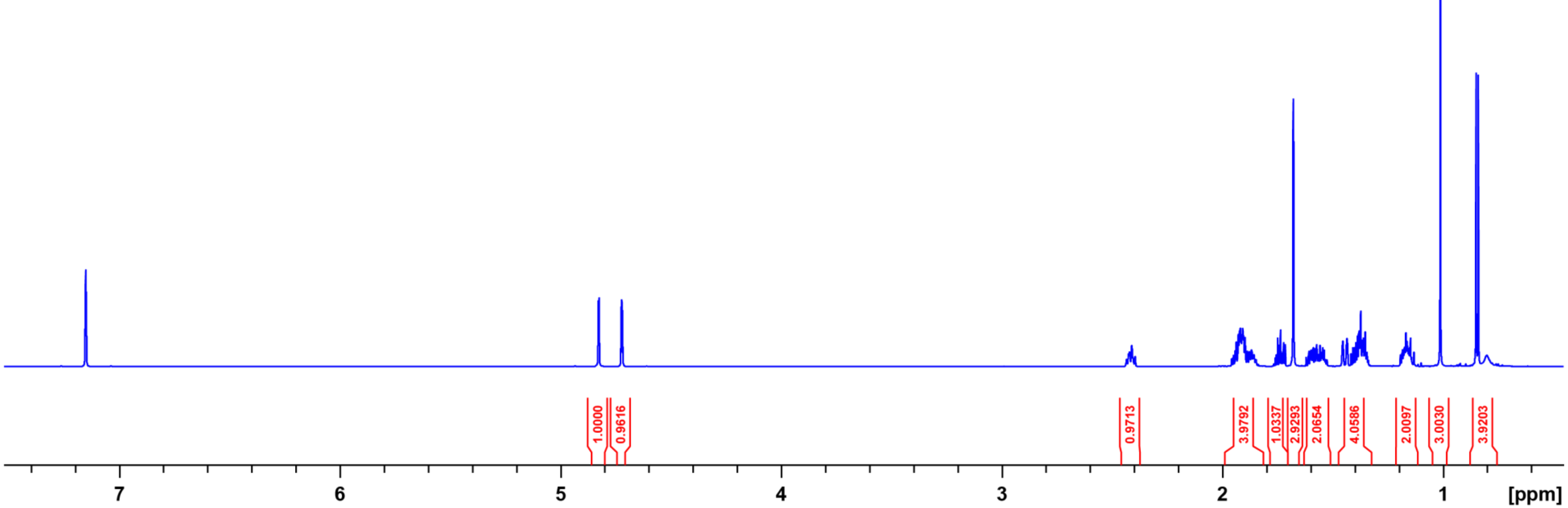

Figure S14: ^{1}H NMR spectrum of **12** (700 MHz, C_6D_6).

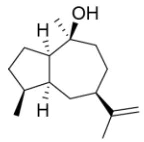

12

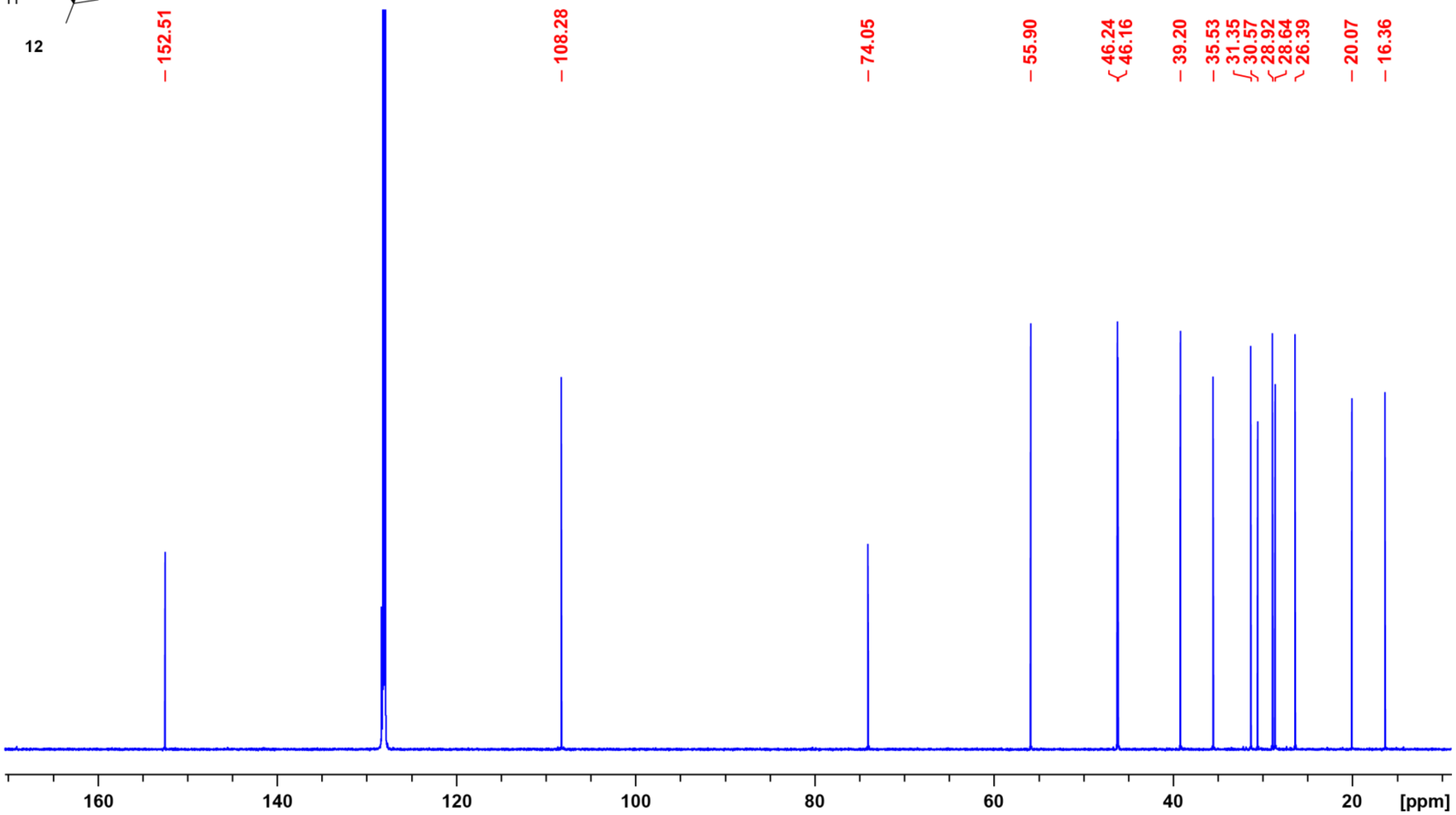

Figure S15: ^{13}C NMR spectrum of **12** (176 MHz, C_6D_6).

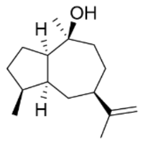

12

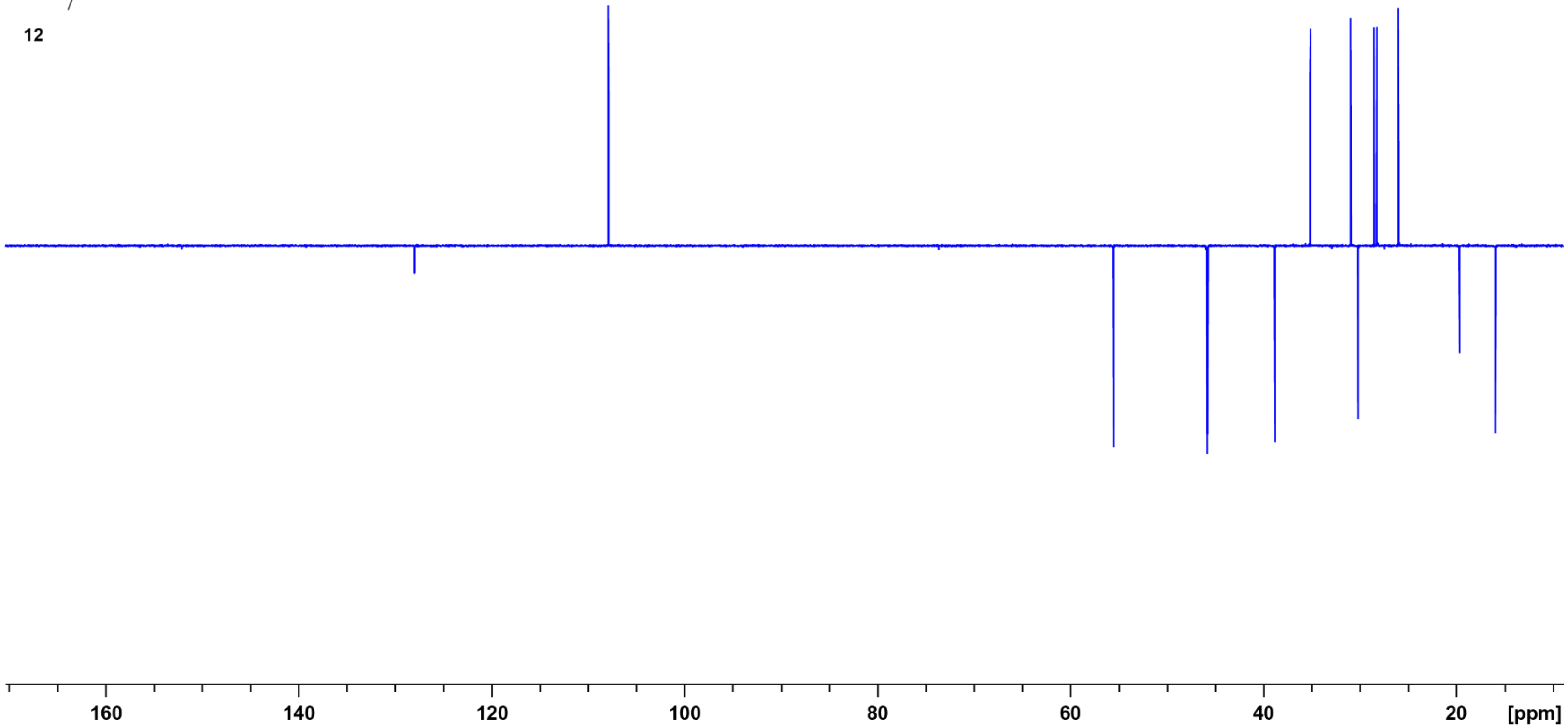

Figure S16: ^{13}C-DEPT spectrum of **12** (176 MHz, C_6D_6).

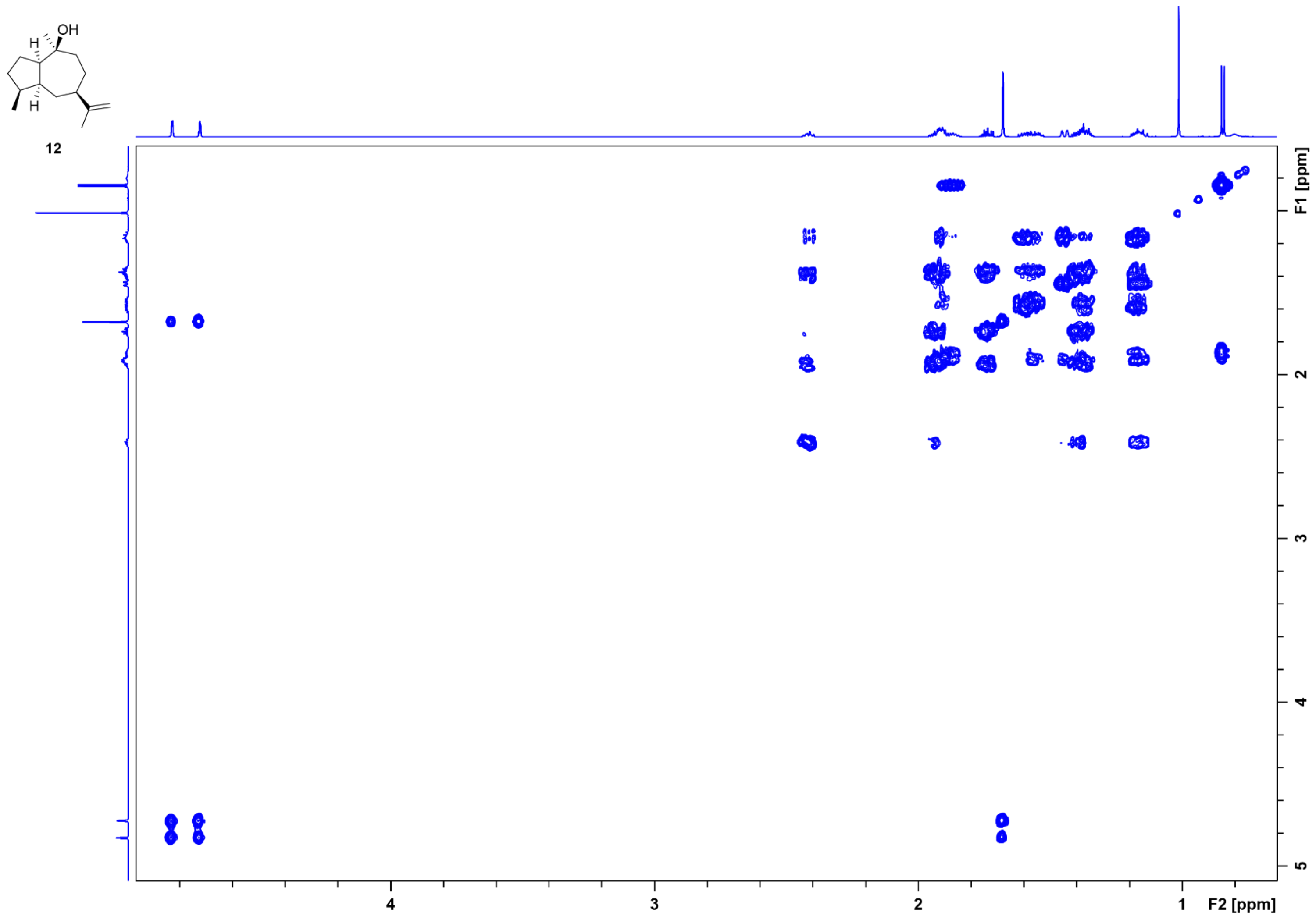

Figure S17: ^1H-^1H-COSY spectrum of **12** (700 MHz, C_6D_6).

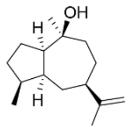

12

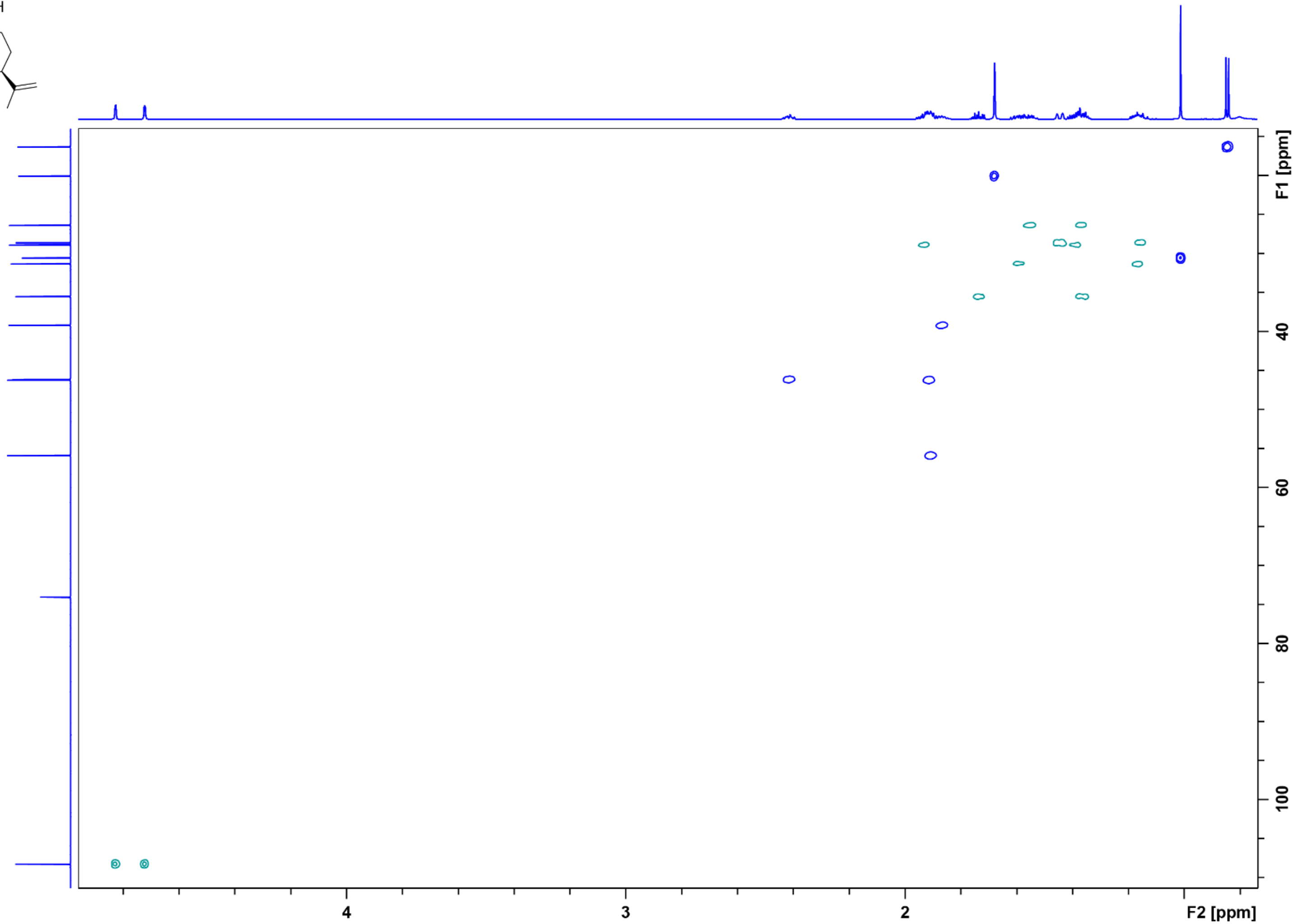

Figure S18: HSQC spectrum of **12** (C_6D_6).

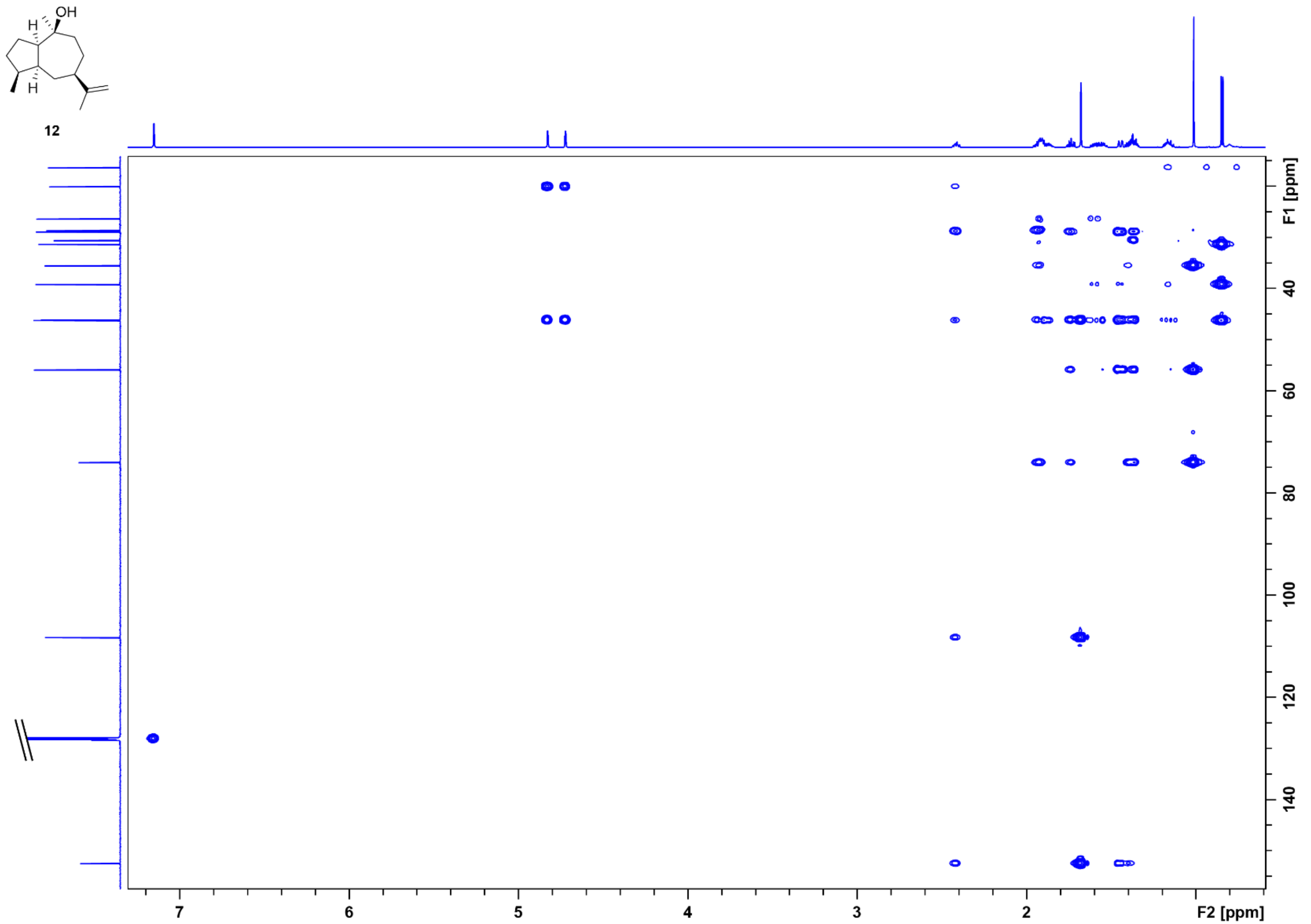

Figure S19: HMBC spectrum of **12** (C_6D_6).

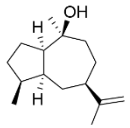

12

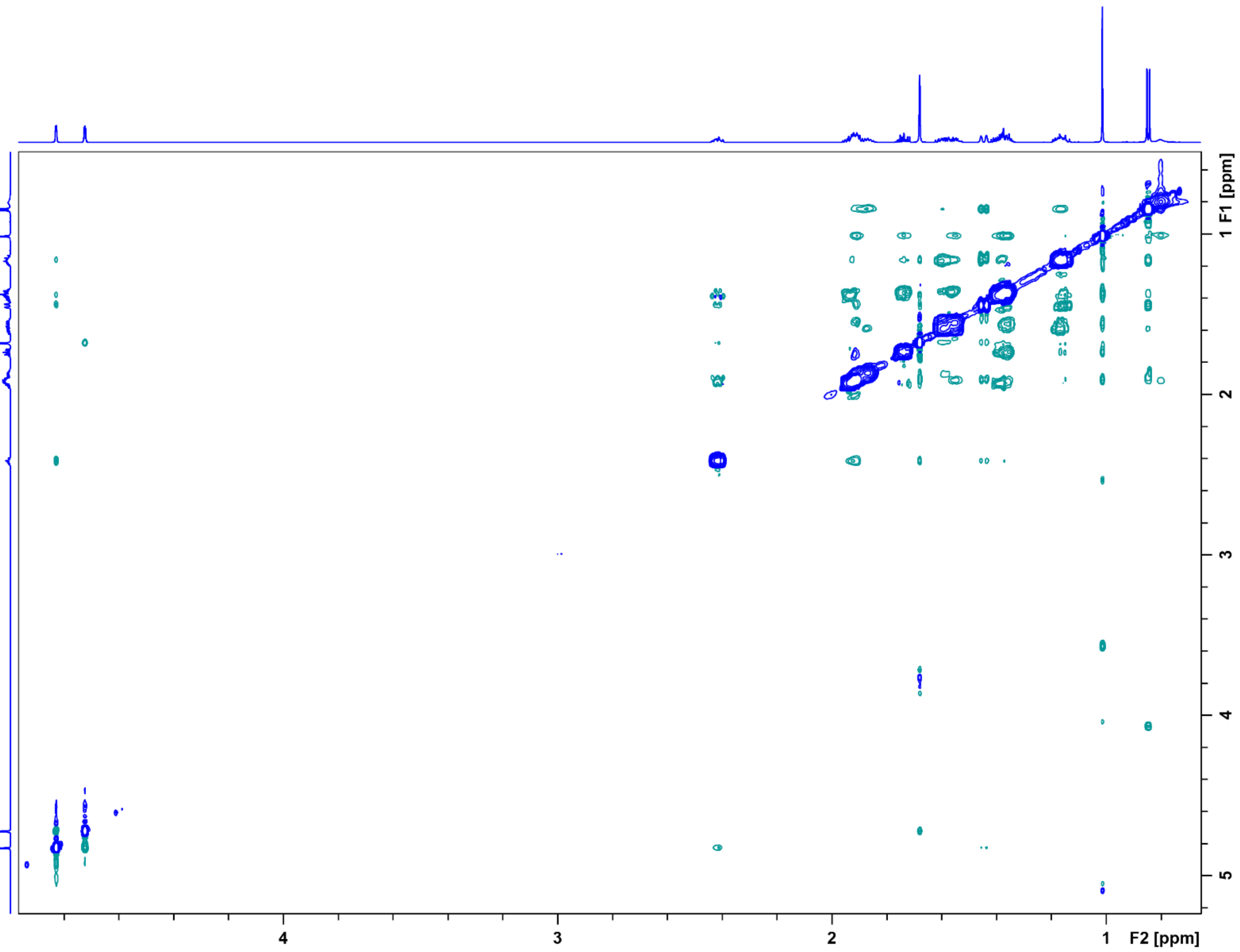

Figure S20: NOESY spectrum of **12** (C_6D_6).

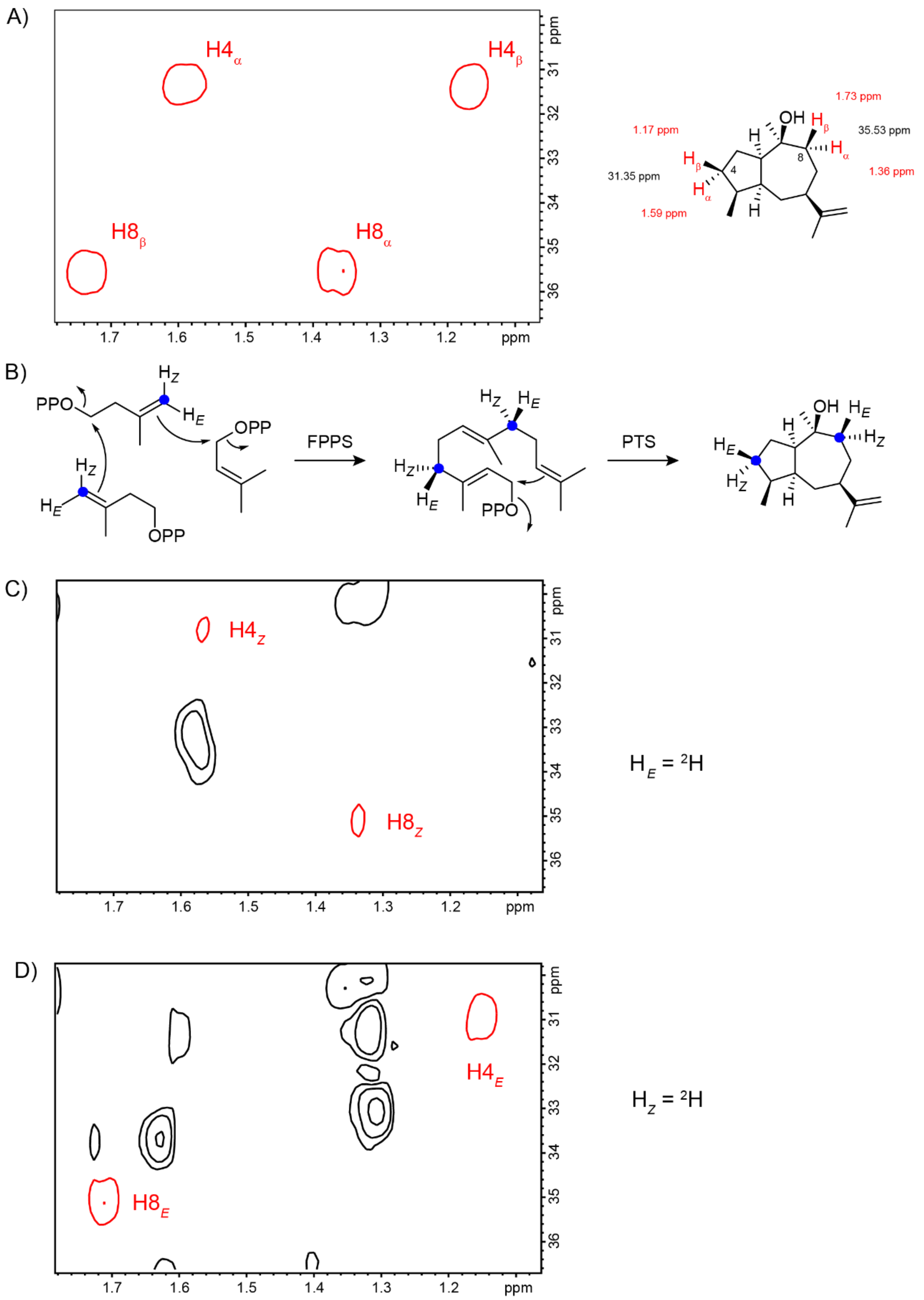

Figure S21: Determination of the absolute configuration of **3**. A) HSQC of unlabelled **12** showing crosspeaks for C4 and C8. HSQC of labelled **12** obtained from B) DMAPP and (*E*)-(4-^{13}C,4-^{2}H)IPP, and C) DMAPP and (*Z*)-(4-^{13}C,4-^{2}H)IPP with FPPS and PTS. The signals for deuterium incorporation are vanished, and because the configurations at deuterated carbons are known, the absolute configuration of **12** can be concluded.

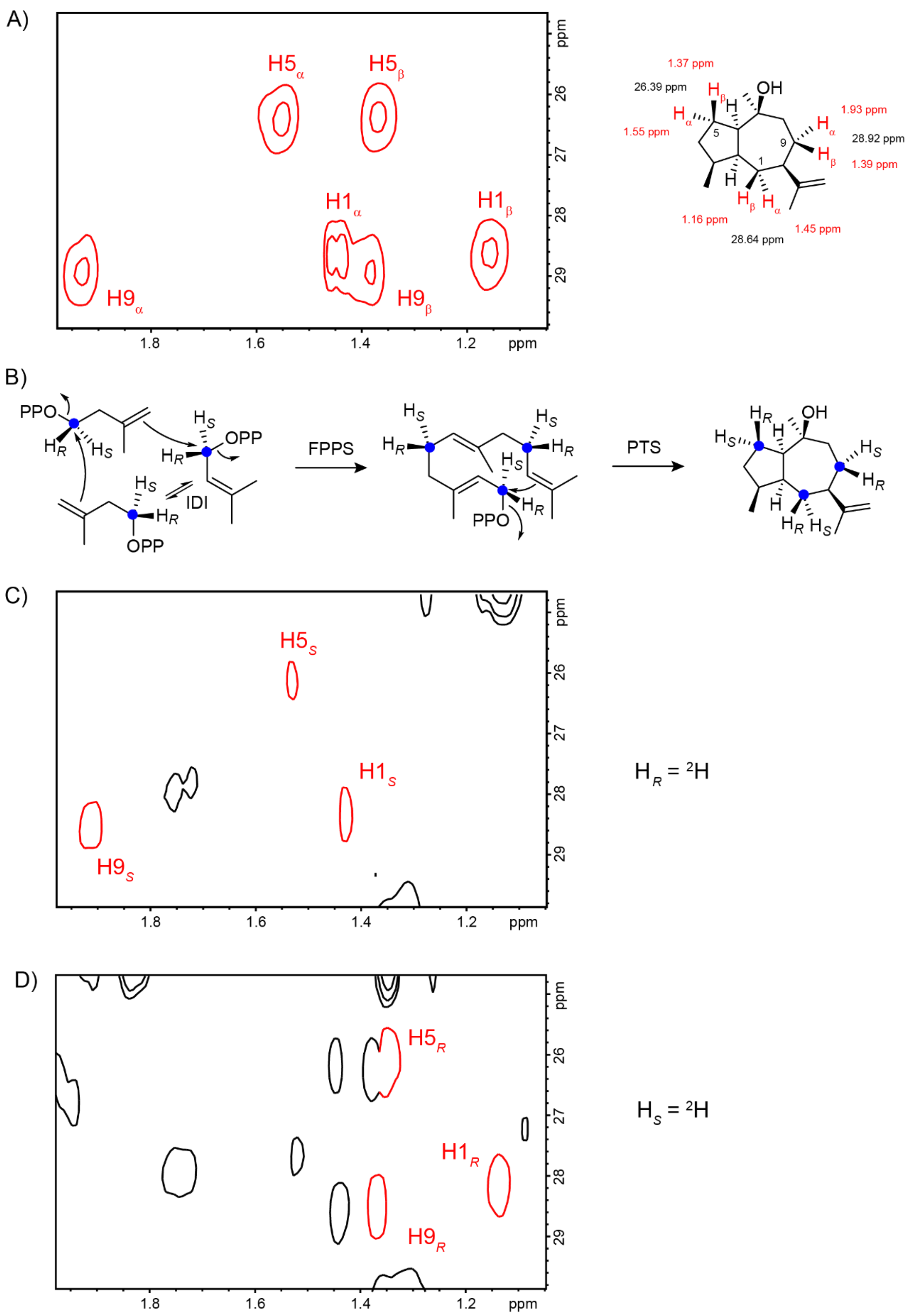

Figure S22: Determination of the absolute configuration of **12**. A) HSQC of unlabelled **12** showing crosspeaks for C1, C5, and C9. HSQC of labelled **12** obtained from B) (*R*)-(1-^{13}C,1-^{2}H)IPP, and C) (*S*)-(1-^{13}C,1-^{2}H)IPP with IDI, FPPS and PTS. The signals for deuterium incorporation are vanished, and because the configurations at deuterated carbons are known, the absolute configuration of **12** can be concluded.

A)

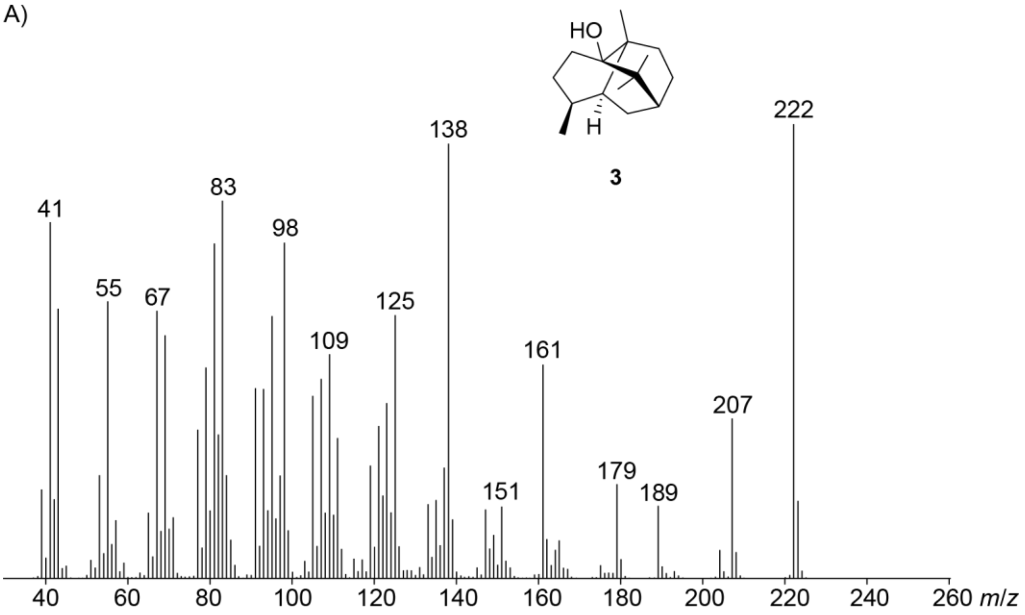

B)

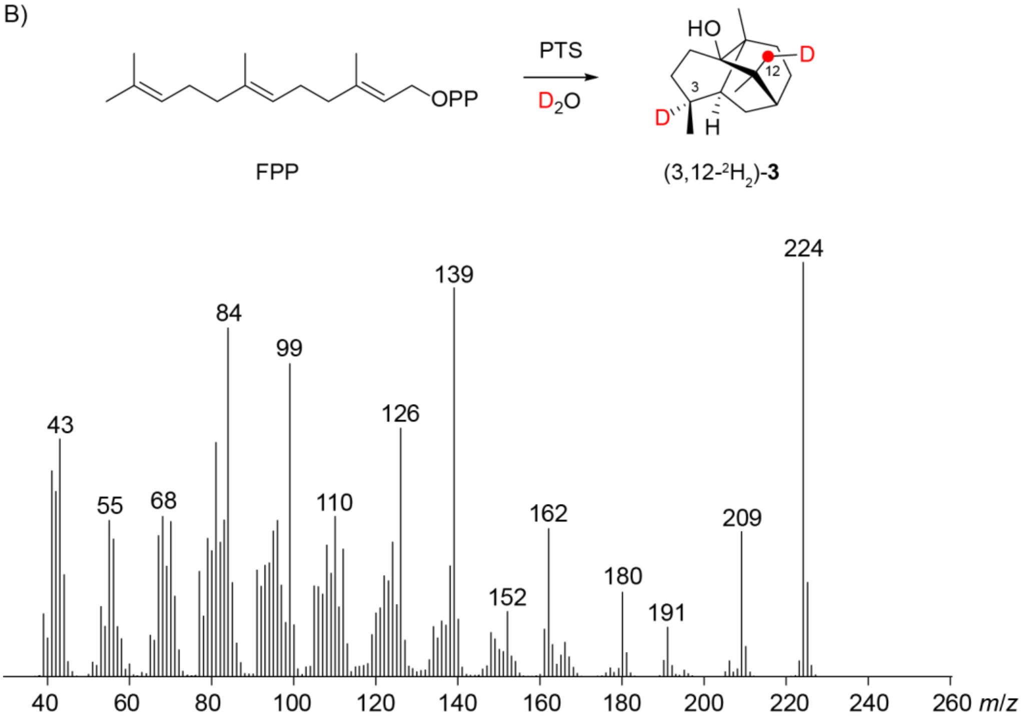

Figure S23: Incubation of FPP with PTS in deuterium oxide buffer. Mass spectra (EI), A) of unlabelled patchoulol (**3**) and B) of labelled **3** from the incubation in D_2O showing incorporation of two deuterium atoms.

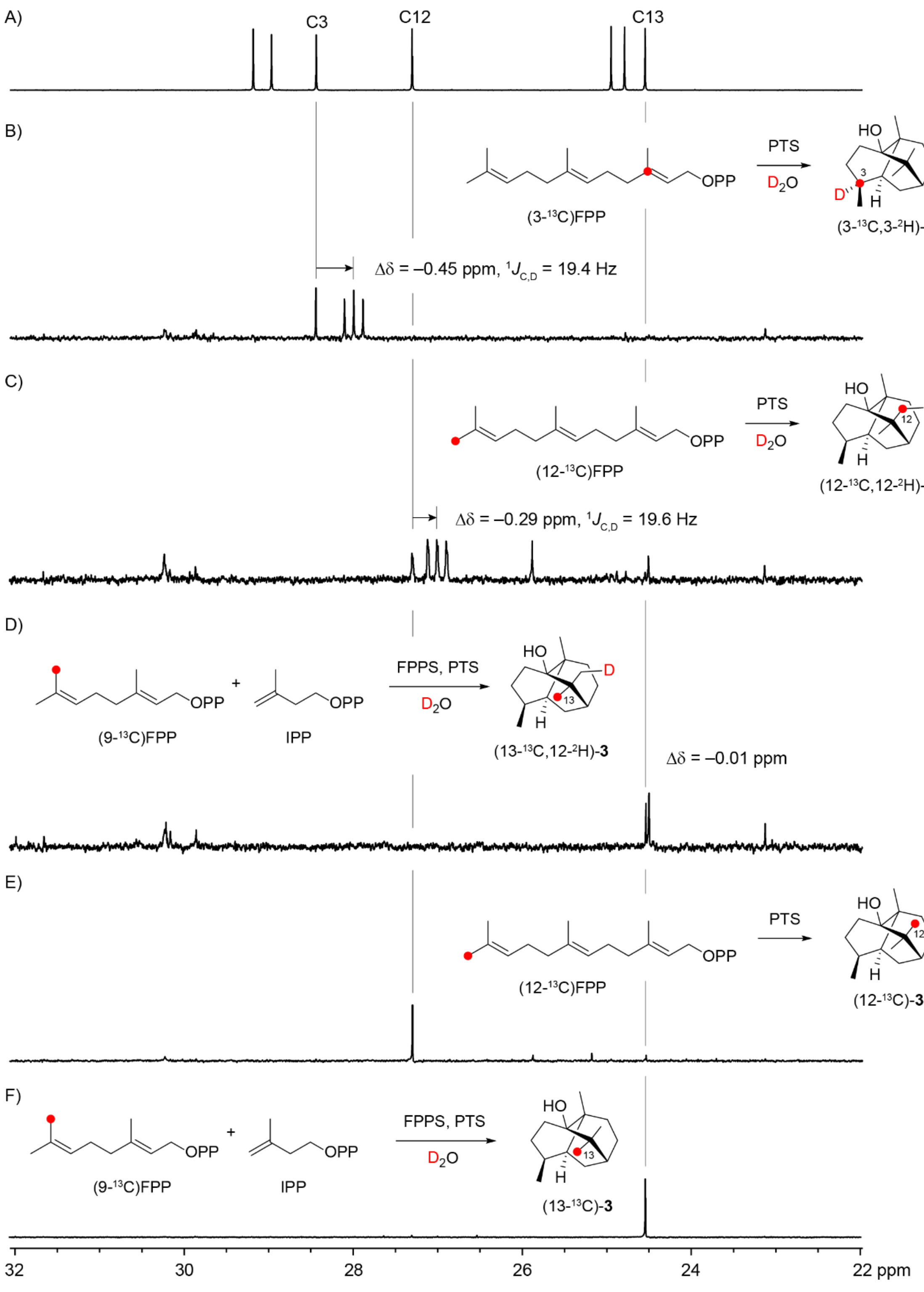

Figure S24: Determination of the sites of deuterium incorporation from the medium into **3** and stereochemical course of the geminal Me groups of FPP. Partial ^{13}C NMR spectra of A) unlabelled **3**, B) (3-^{13}C,3-^{2}H)-**3** obtained from (3-^{13}C)FPP with PTS in D_2O buffer, C) (12-^{13}C,12-^{2}H)-**3** obtained from (12-^{13}C)FPP with PTS in D_2O buffer, D) (13-^{13}C,12-^{2}H)-**3** obtained from (9-^{13}C)GPP and IPP with FPPS and PTS in D_2O buffer, E) (12-^{13}C)-**3** obtained from (12-^{13}C)FPP with PTS, and F) (13-^{13}C)-**3** obtained from (9-^{13}C)GPP and IPP with FPPS and PTS.

A)

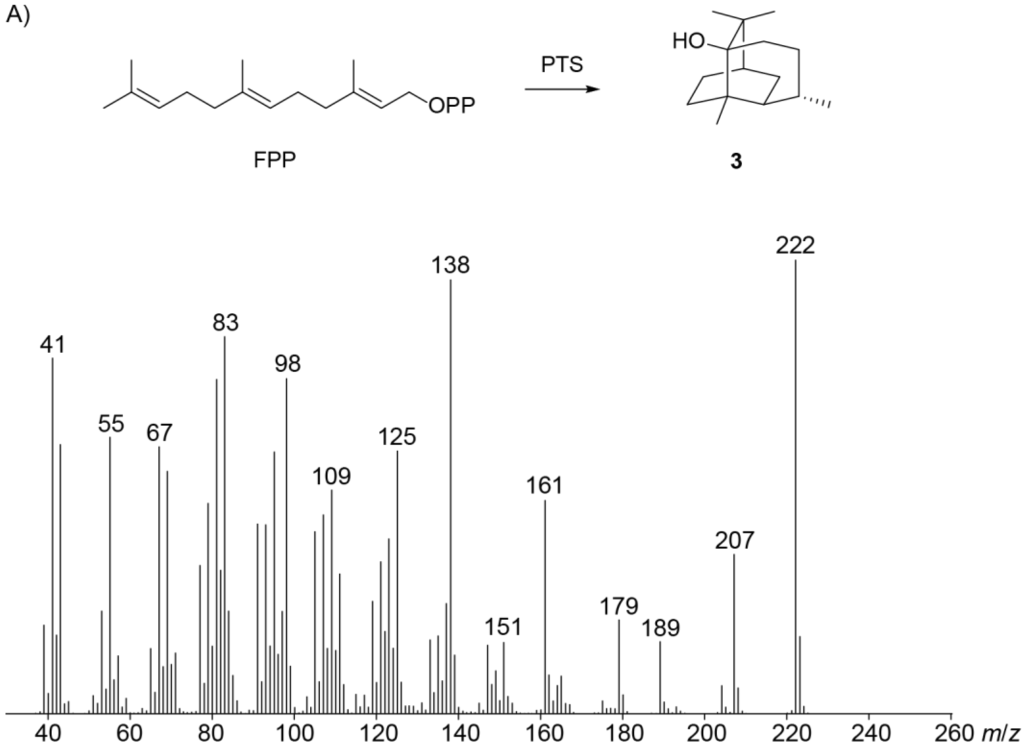

B)

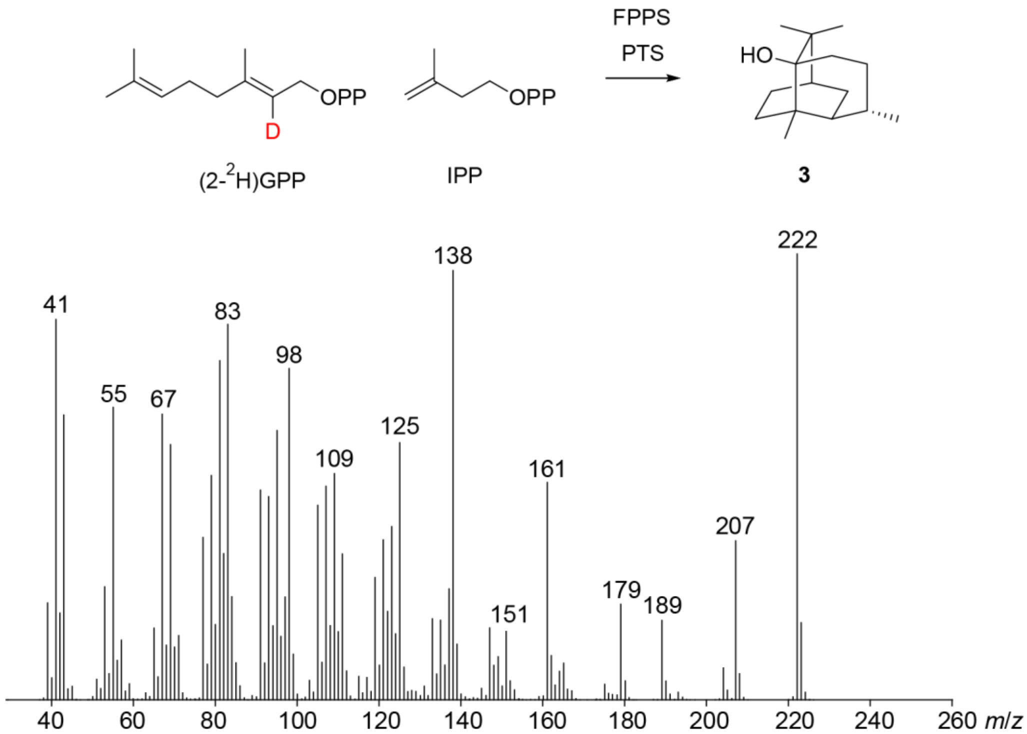

Figure S25: EI mass spectra of A) **3** obtained from FPP and B) **3** obtained from an incubation of (2-^{2}H)GPP and IPP with FPPS and PTS, showing complete loss of deuterium.

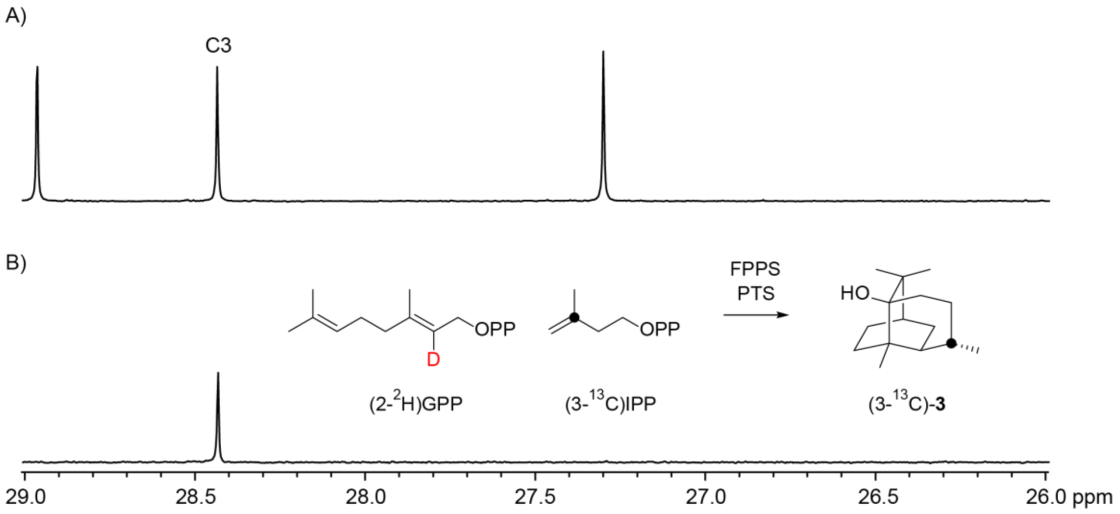

Figure S26: Partial ^{13}C NMR spectra of A) unlabelled **3** and B) labelled (3-^{13}C)-**3** obtained from an incubation of (2-^{2}H)GPP and (3-^{13}C)IPP with FPPS and PTS. The absence of a triplet peak in B) indicates that the 1,4-hydride shift from **C** to **D** (Scheme 1 of main text) or the 1,3-hydride shift from **H** to **J** (Scheme 3 of main text) does not occur.

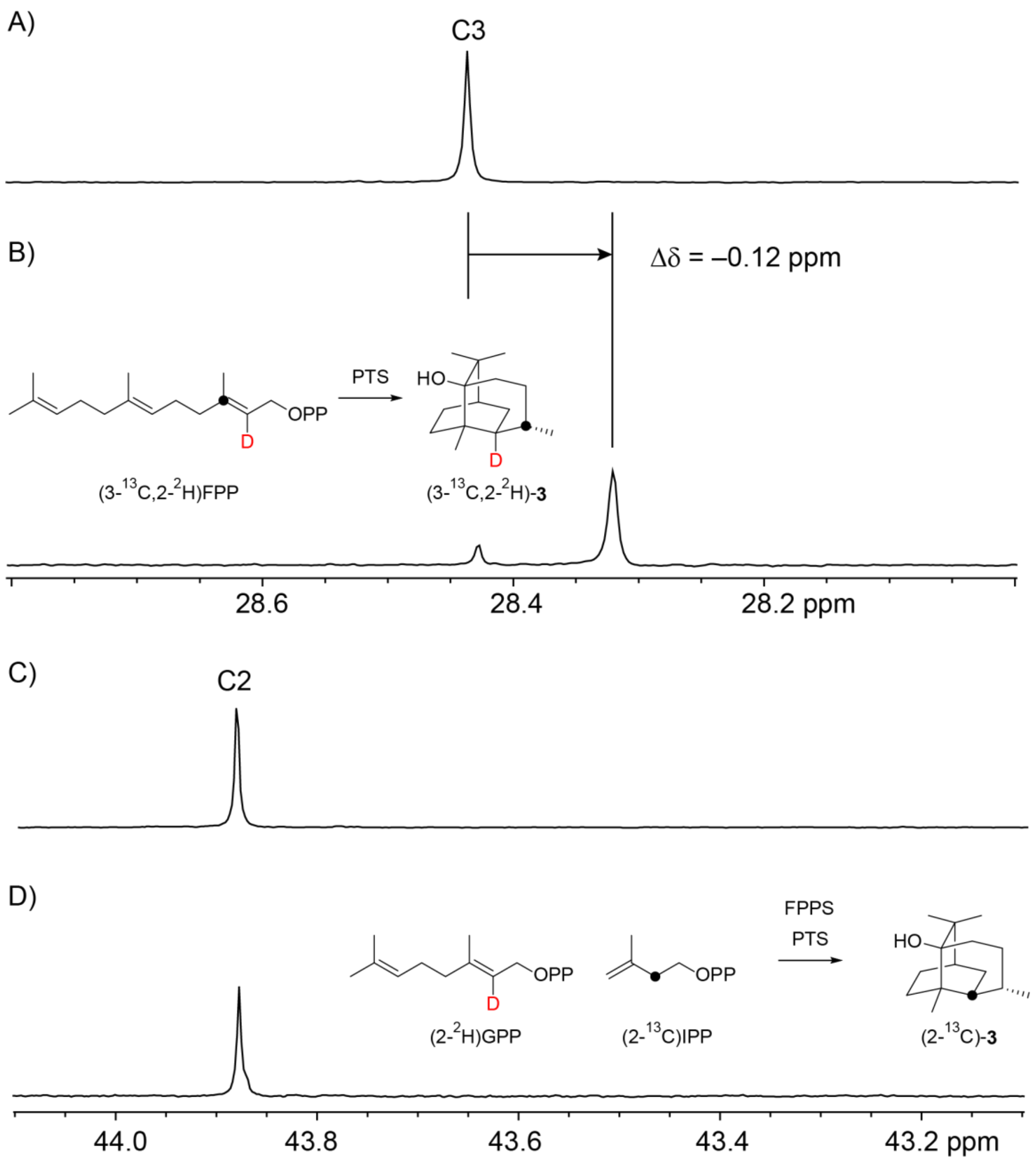

Figure S27: Partial ^{13}C NMR spectra A) of unlabelled **3** showing the region for C3, and B) of labelled **3** obtained from (3-^{13}C,2-^{2}H)FPP with PTS. The small upfield shift indicates deuterium bound to a neighbouring carbon of C3. Partial ^{13}C NMR spectra C) of unlabelled **3** showing the region for C2, and D) of labelled **3** obtained from (2-^{2}H)GPP and (2-^{13}C)IPP with FPPS and PTS. The absence of triplet signals for the labelled carbons in B) and D) excludes a series of 1,2-hydride shifts as shown in Scheme 3 of main text (from **H** via **I** to **J**).

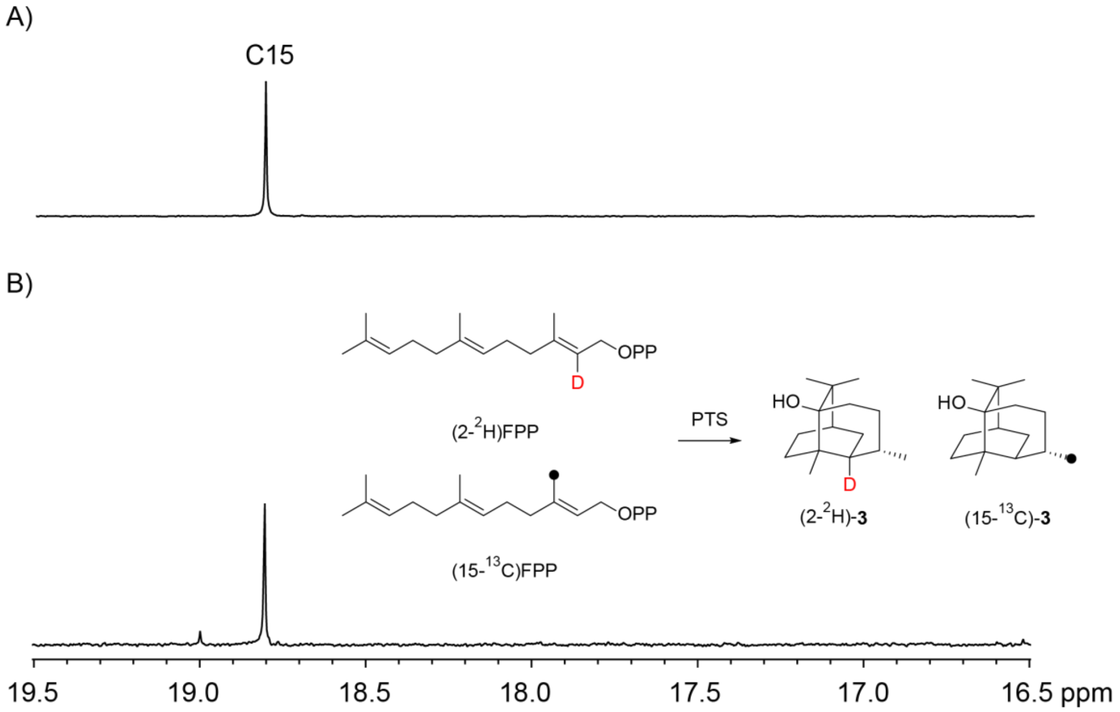

Figure S28: Partial ^{13}C NMR spectra A) of unlabelled **3** showing the region for C15, and B) of labelled **3** obtained from the mixed substrates (2-^{2}H)FPP and (15-^{13}C)FPP with PTS. The absence of a triplet for C15 in B) excludes the intermolecular deuterium transfer from (2-^{2}H)FPP to (15-^{13}C)FPP.

DFT calculations

All computed structures are geometry optimised without restrictions and are characterised as minima or as transition state structures by frequency analyses, also providing Gibbs-corrections, using the B97D3/6-31g(d,p) method with the density fitting approximation for s- and p-functions, including Grimme's empirical D3-dispersion correction [12] in Gaussian16 [13].

For improved single point energies, the mPW1PW91 functional is applied with the 6-311+G(d,p) basis set without density fitting and the ultra-fine integration grid, as this method has shown to be very reliable for examining carbocation cyclisation rearrangement reactions [14-17].

The Gibbs-corrections include an entropic quasi-harmonic treatment with a frequency cut-off value of 100.0 wavenumbers, according to Grimme, using a mixture of RRHO and free-rotor vibrational entropies [18,19].

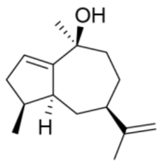

17

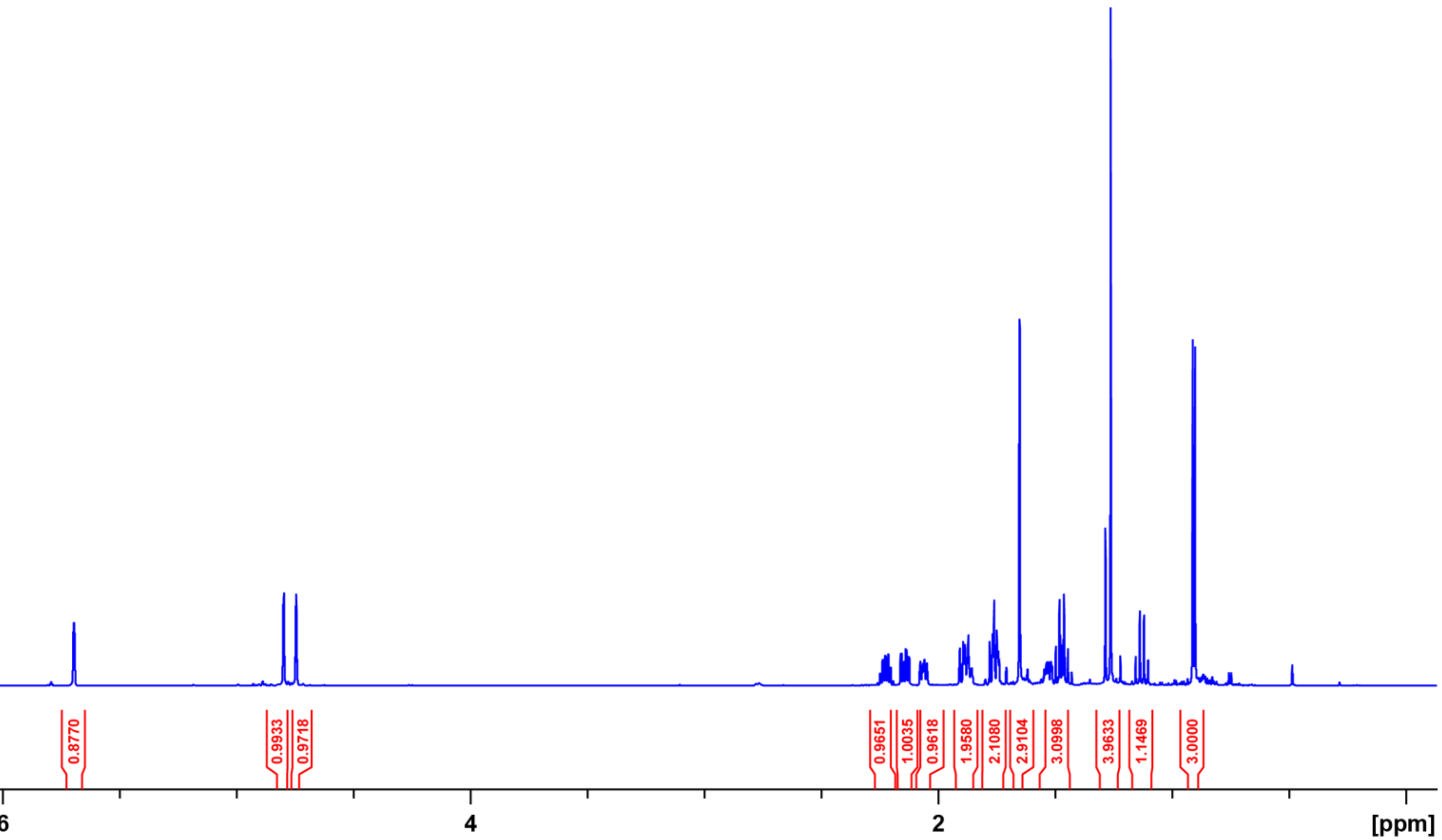

Figure S29: ^{1}H NMR spectrum of **17** (700 MHz, C_6D_6).

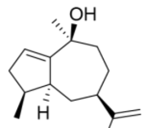

17

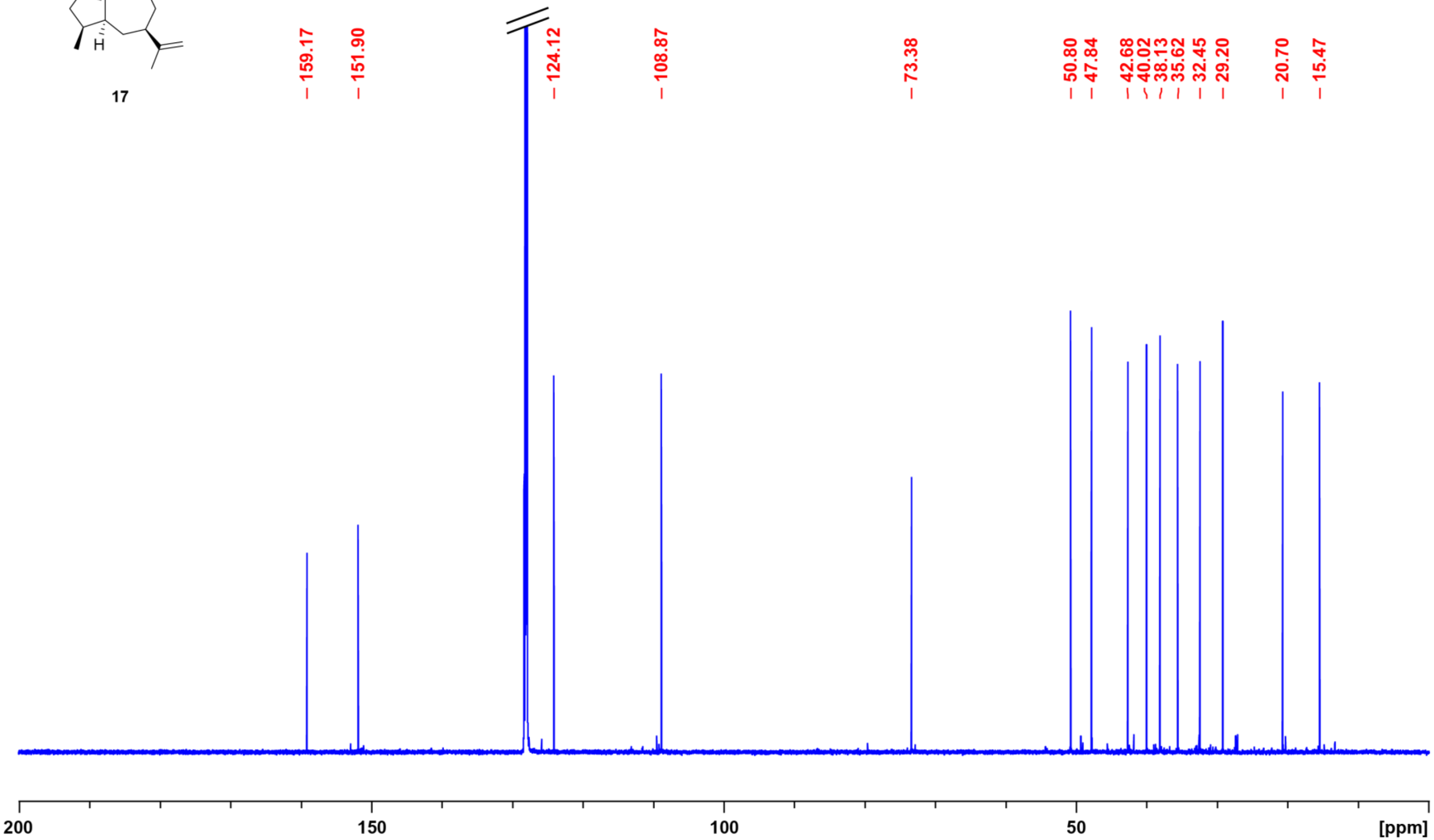

Figure S30: ^{13}C NMR spectrum of **17** (176 MHz, C_6D_6).

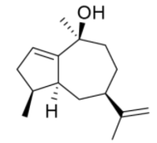

17

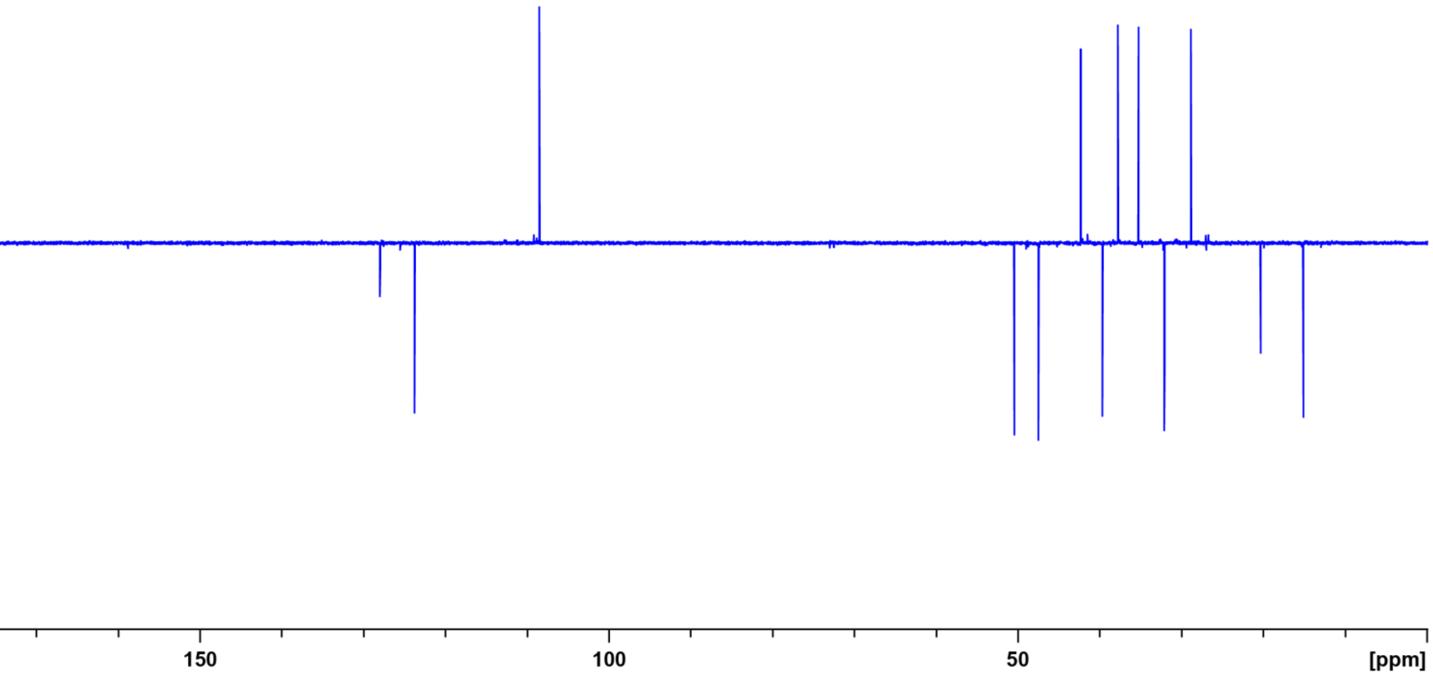

Figure S31: ^{13}C-DEPT spectrum of **17** (176 MHz, C_6D_6).

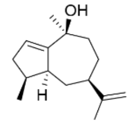

17

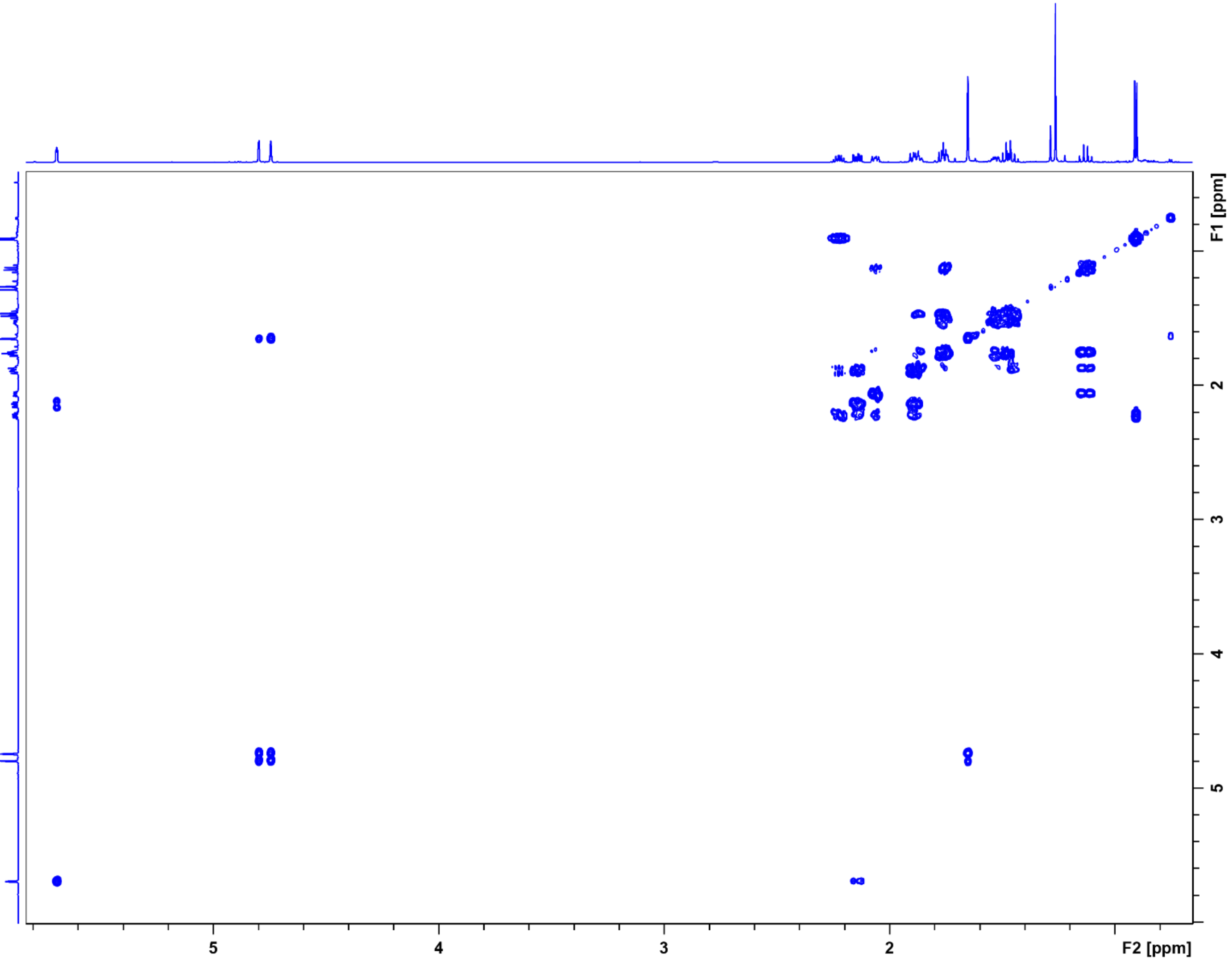

Figure S32: ^{1}H-^{1}H-COSY spectrum of **17** (700 MHz, C_6D_6).

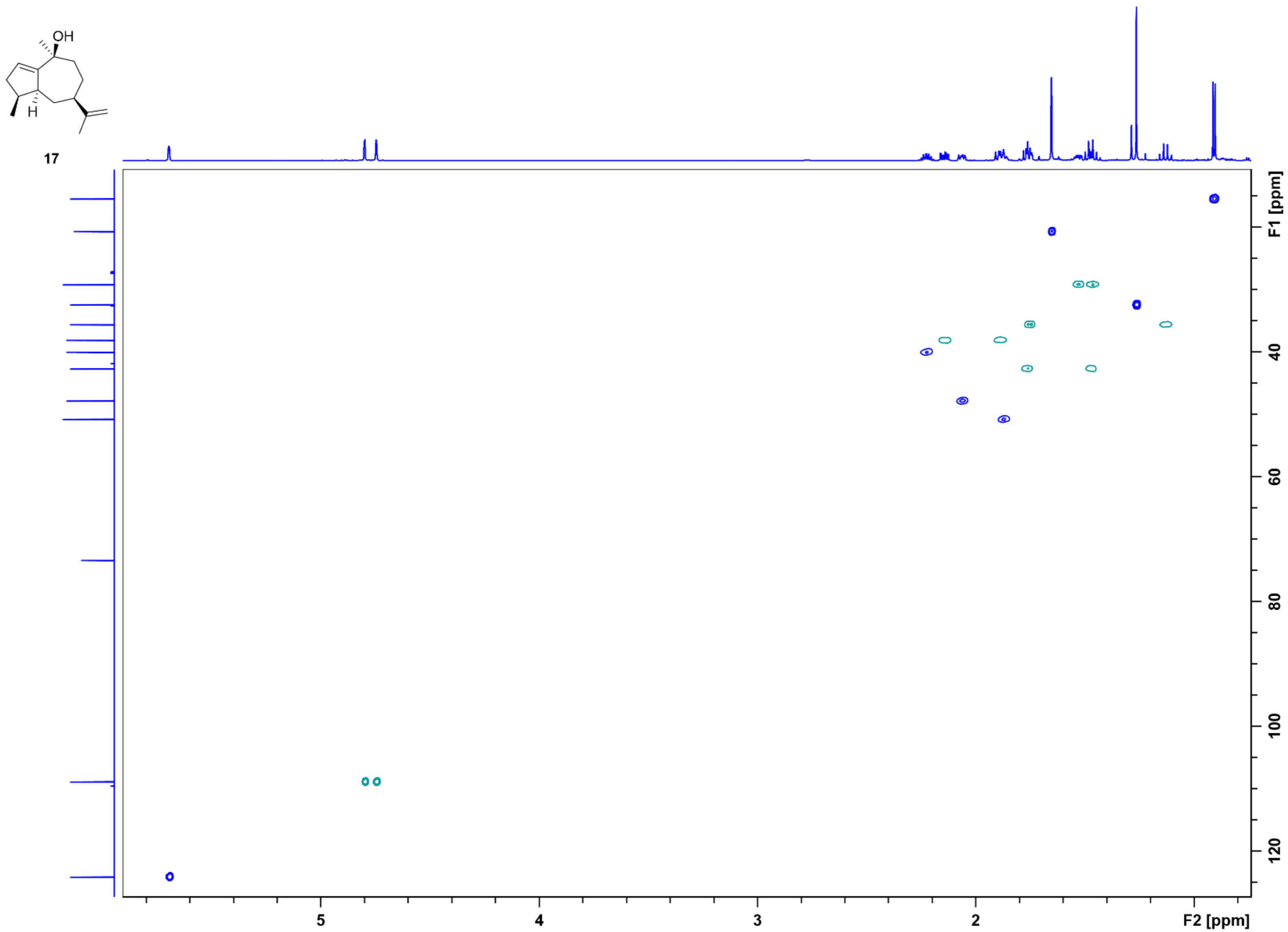

Figure S33: HSQC spectrum of **17** (C_6D_6).

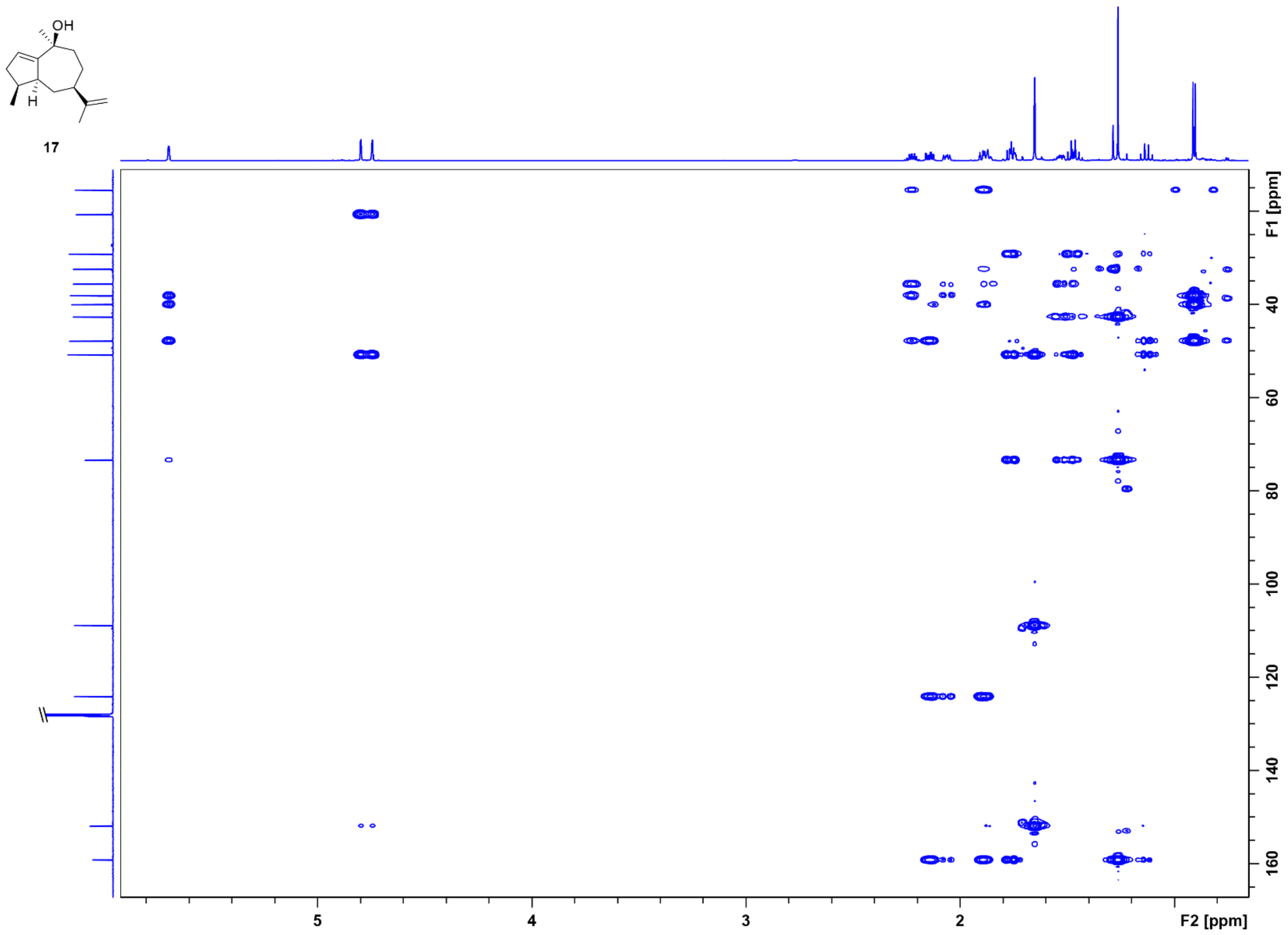

Figure S34: HMBC spectrum of **17** (C_6D_6).

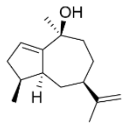

17

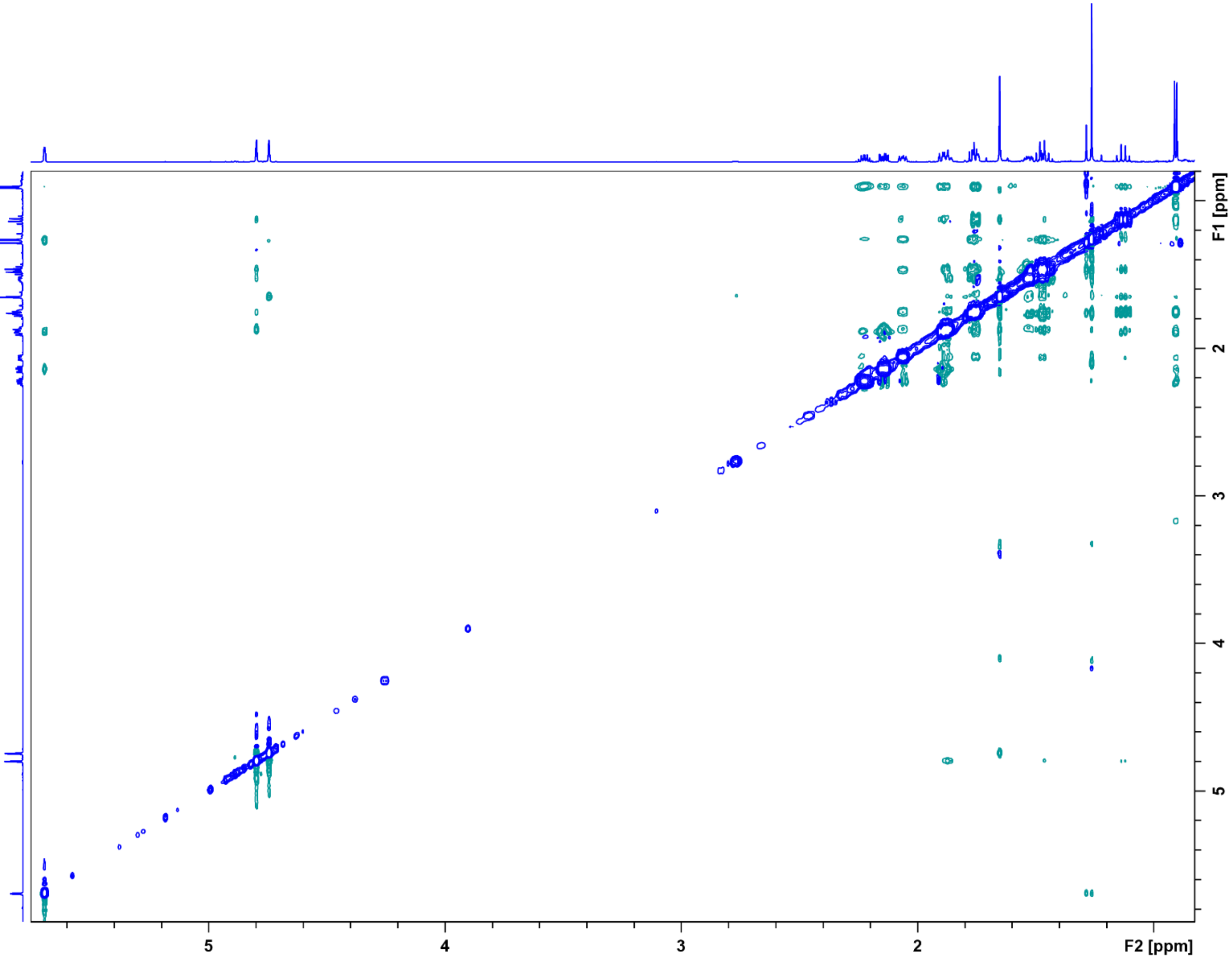

Figure S35: NOESY spectrum of **17** (C_6D_6).

References

1. Fulmer, G. R.; Miller, A. J. M.; Sherden, N. H.; Gottlieb, H. E.; Nudelman, A.; Stoltz, B. M.; Bercaw, J. E.; Goldberg, K. I. *Organometallics,* **2010,** *29,* 2176–2179.
2. Blessing, R. H. *Acta Crystallogr. A,* **1995,** *51,* 33–38.
3. Sheldrick, G. M. *Acta Crystallogr. Sect. C Struct. Chem.,* **2015,** *71,* 3–8.
4. Hooft, R. W. W.; Straver, L. H.; Spek, A. L. *J. Appl. Crystallogr.,* **2008,** *41,* 96–103.
5. Lauterbach, L.; Rinkel, J.; Dickschat, J. S. *Angew. Chem. Int. Ed.,* **2018,** *57,* 8280–8283.
6. Rabe, P.; Rinkel, J.; Nubbemeyer, B.; Köllner, T. G.; Chen, F.; Dickschat, J. S. *Angew. Chem. Int. Ed.,* **2016,** *55,* 15420–15423.
7. Rinkel, J.; Dickschat, J. S. *Org. Lett.,* **2019,** *21,* 2426–2429.
8. Rabe, P.; Barra, L.; Rinkel, J.; Riclea, R.; Citron, C. A.; Klapschinski, T. A.; Janusko, A.; Dickschat, J. S. *Angew. Chem. Int. Ed.,* **2015,** *54,* 13448–13451.
9. Bian, G.; Rinkel, J.; Wang, Z.; Lauterbach, L.; Hou, A.; Yuan, Y.; Deng, Z.; Liu, T.; Dickschat, J. S. *Angew. Chem. Int. Ed.,* **2018,** *57,* 15887–15890.
10. Rabe, P.; Rinkel, J.; Dolja, E.; Schmitz, T.; Nubbemeyer, B.; Luu, T. H.; Dickschat, J. S. *Angew. Chem. Int. Ed.,* **2017,** *56,* 2776–2779.
11. Klapschinski, T. A.; Rabe, P.; Dickschat, J. S. *Angew. Chem. Int. Ed.,* **2016,** *55,* 10141–10144.
12. Grimme, S.; Ehrlich, S.; Goerigk, L. *J. Comp. Chem.,* **2011,** *32,* 1456–1465.
13. Frisch, M. J. et al. Gaussian 16, Revision B.01, Gaussian, Inc., Wallingford CT, **2016**.
14. Hong, Y. J.; Tantillo, D. J. *J. Org. Chem.,* **2018,** *83,* 3780–3793.
15. Adamo, C.; Barone, V. *J. Chem. Phys.,* **1998,** *108,* 664–675.
16. Matsuda, S. P. T.; Wilson, W. K.; Xiong, Q. *Org. Biomol. Chem.,* **2006,** *4,* 530–543.
17. Lauterbach, L.; Goldfuss, B.; Dickschat, J. S. *Angew. Chem. Int. Ed.,* **2020,** *59,* 11943–11947.
18. Grimme, S.; *Chem. Eur. J.,* **2012,** *18,* 9955–9964.
19. Luchini, G.; Alegre-Requena, J. V.; Guan, Y.; Funes-Ardoiz, I.; Paton, R. S. GoodVibes v3.0.1, **2019**.
